# Epigenetic reactivation of tumor suppressor genes with CRISPRa technologies as precision therapy for hepatocellular carcinoma

**DOI:** 10.1186/s13148-023-01482-0

**Published:** 2023-04-29

**Authors:** Agustin Sgro, Joseph Cursons, Charlene Waryah, Eleanor A. Woodward, Momeneh Foroutan, Ruqian Lyu, George C. T. Yeoh, Peter J. Leedman, Pilar Blancafort

**Affiliations:** 1 Cancer Epigenetics Group, The Harry Perkins Institute of Medical Research, Nedlands, Perth, WA 6009 Australia; 2 Centre for Medical Research, The University of Western Australia, Perth, WA 6009 Australia; 3 School of Human Sciences, The University of Western Australia, Crawley, Perth, WA 6009 Australia; 4 Biomedicine Discovery Institute and the Department of Biochemistry and Molecular Biology, Monash University, Clayton, VIC 3800 Australia; 5 Bioinformatics and Cellular Genomics, St Vincent’s Institute of Medical Research, Fitzroy, Melbourne, VIC 3065 Australia; 6 Melbourne Integrative Genomics/School of Mathematics and Statistics, Faculty of Science, The University of Melbourne, Royal Parade, Parkville, VIC 3010 Australia; 7 School of Molecular Sciences, University of Western Australia, Crawley, Perth, WA 6009 Australia; 8 Laboratory for Cancer Medicine, Harry Perkins Institute of Medical Research, QEII Medical Centre, 6 Verdun St, Nedlands, Perth, WA 6009 Australia; 9 School of Medicine and Pharmacology, The University of Western Australia, Crawley, Perth, WA 6009 Australia

**Keywords:** CRISPRa, Genome multiplexing, Epigenetic editing toolbox, Tumor suppressor genes, Hepatocellular carcinoma, Epigenetic drugs, Precision medicine

## Abstract

**Background:**

Epigenetic silencing of tumor suppressor genes (TSGs) is a key feature of oncogenesis in hepatocellular carcinoma (HCC). Liver-targeted delivery of CRISPR-activation (CRISPRa) systems makes it possible to exploit chromatin plasticity, by reprogramming transcriptional dysregulation.

**Results:**

Using The Cancer Genome Atlas HCC data, we identify 12 putative TSGs with negative associations between promoter DNA methylation and transcript abundance, with limited genetic alterations. All HCC samples harbor at least one silenced TSG, suggesting that combining a specific panel of genomic targets could maximize efficacy, and potentially improve outcomes as a personalized treatment strategy for HCC patients. Unlike epigenetic modifying drugs lacking locus selectivity, CRISPRa systems enable potent and precise reactivation of at least 4 TSGs tailored to representative HCC lines. Concerted reactivation of *HHIP*, *MT1M*, *PZP*, and *TTC36* in Hep3B cells inhibits multiple facets of HCC pathogenesis, such as cell viability, proliferation, and migration.

**Conclusions:**

By combining multiple effector domains, we demonstrate the utility of a CRISPRa toolbox of epigenetic effectors and gRNAs for patient-specific treatment of aggressive HCC.

**Supplementary Information:**

The online version contains supplementary material available at 10.1186/s13148-023-01482-0.

## Background

Hepatocellular carcinoma (HCC) is the most prevalent form of liver cancer and the third most lethal cancer worldwide [1]. Chronic liver inflammation progressively induces fibrosis leading to cirrhosis and ultimately, in a small percentage of people, HCC [2]. During this process, hepatocytes acquire multiple genetic and epigenetic alterations [3, 4]. Furthermore, a diverse range of etiologies such as viral hepatitis B and C infections, the metabolic syndrome, diabetes, obesity, non-alcoholic steatohepatitis, and chronic alcohol consumption contribute to the pathogenesis and development of HCC [5].

Early stages of HCC are treated with potentially curative locoregional therapies; however, high rates of recurrence (70%) 5 years post-hepatic resection constitute a serious impasse, and adjuvant treatments to prevent relapse represent a still unmet medical need [6]. There are six approved systemic therapies for the management of advanced unresectable HCC. First-line treatments include the multi-kinase inhibitors, such as sorafenib and lenvatinib, and the combination of atezolizumab (anti-PD-L1) and bevacizumab (anti-VEGF-A) antibodies. For non-responders, the second-line single-agent regimens consist of multi-kinase inhibitors, such as regorafenib, cabozantinib, or ramucirumab (anti-VEGFR2 antibody). Emerging FDA-approved immune therapies include checkpoint blockades, such as nivolumab and pembrolizumab (anti-PD-1), and the combination of nivolumab with ipilimumab (anti-CTLA-4) antibodies [7]. While providing 8 to 19.2 months of survival benefit [5], both resistance to these treatments and limited tolerability can lead to severe and, in some cases, unmanageable adverse events in HCC patients [5, 8–11]. Thus, there is a pressing clinical need for new targeted and potentially more tolerable therapies for the treatment of HCC.

Recent “multi-omics” data from 363 liver cancer patients from The Cancer Genome Atlas (TCGA) identified three integrative Cluster (iClust) HCC subtypes based on the integration of several molecular features, including transcript abundance, copy number and sequence variations, and DNA methylation [12]. Clinically, the iClust1 subtype comprises patients of younger age, female gender, and Asian ethnicity. These are high-grade tumors associated with macrovascular invasion and the worst survival outcomes. The iClust2 includes low-grade HCC with less microvascular invasion and overall better prognosis than the iClust1 and iClust3 subtypes. Lastly, iClust3 tumors exhibit higher frequencies of chromosomal instability, *TP53* mutations, and DNA hypomethylation throughout the genome. These genomic studies also highlighted potential tumor suppressor genes (TSGs) presenting DNA promoter hypermethylation concurrent with gene silencing. While some TSGs are already known to play a key role in HCC pathogenesis, including the cell cycle regulator *CDKN2A* [12–14]*,* some less well-characterized targets have been found selectively silenced in specific iClust HCC subtypes. However, the causative functional role of these TSGs during HCC pathogenesis remains unknown. Thus, genome engineering technologies able to specifically and selectively reactivate these TSGs could facilitate the functional interrogation of these loci as potential drivers of HCC. Importantly, these technologies also represent a new generation of precision oncology approaches for the personalized treatment of HCC.

Reactivation of TSGs has previously been achieved with inhibitors of epigenetic enzymes that either deposit silencing marks in the DNA and associated histones, such as DNA methyltransferases (DNMTs), including 5-aza-2′-deoxycytidine (decitabine), or that erase activating marks, such as histone deacetylases (HDACs), including suberoylanilide hydroxamic acid (vorinostat). However, potential limitations of these regimes include high toxicities after prolonged treatment, with the eventual development of drug resistance. These toxicities have been correlated with genome-wide effects due to the lack of locus selectivity of these treatments [15–17].

Clustered Regularly Interspaced Short Palindromic Repeats/CRISPR-associated protein 9 (CRISPR/Cas9) adapted for epigenetic editing represents an emerging technology to reactivate genes with high selectivity, thereby enabling functional interrogation of TSGs. In CRISPR-activation (CRISPRa) systems, a catalytically dead nuclease from *Streptococcus pyogenes*, *Sp*dCas9, is fused to one or more activator domains or epigenetic effector domains (EDs). These fusions are directed or targeted to a genomic region by co-expression of a 20-nucleotide (nt) guide RNA (gRNA). This versatile system has been exploited for specific re-expression of several loci by expression of multiple gRNAs targeting different TSGs, such as *MASPIN* in lung cancer cells [18], *RPRM* in gastric cancer cells [18], *PTEN* in triple-negative breast cancer and melanoma [19], *DKK3* in prostate cancer [20], and *CCN6* in a mouse model of breast cancer [21].

In the context of HCC, CRISPR/Cas9 technologies have been employed for genome-scale knockout screens to identify TSGs, such as *ADAMTSL3* and *PTEN* [22]. Similarly, a CRISPRa screening library has enabled the identification of key drivers of sorafenib resistance, such as *PHGDH* [23] and *LRP8* [24]. However, to the best of our knowledge, CRISPRa platforms have not been adopted for rational and simultaneous targeting of multiple TSGs silenced in clinical specimens of HCC, particularly in the aggressive subtypes of HCC.

In this study, we bioinformatically analyzed a panel of 12 candidate TSGs (*BCO2*, *CDKN2A*, *CPS1*, *HHIP*, *miR-122-5p*, *MT1E*, *MT1M*, *PSAT1*, *PTGR1*, *PZP*, *TMEM106A*, and *TTC36*) that are epigenetically silenced and under-expressed in HCC tumor samples compared to normal tissue. By investigating a panel of epigenetic effector domains and gRNAs, we demonstrate that CRISPRa systems strongly and selectively activate up to four different TSGs in representative HCC cell lines harboring hypermethylated and silenced genes. Comparison of the CRISPRa system with epi-drugs (decitabine and vorinostat) confirmed the superior locus selectivity of gRNA systems relative to currently approved epigenetic inhibitors. Our study outlines a customizable epigenetic editing toolkit to reactivate “at will” multiple candidate TSGs in HCC patients.

## Results

### Associations between RNA abundance, promoter DNA hypermethylation, and copy number variation identified a 12-tumor suppressor gene panel

To functionally interrogate multiple TSGs and in a wide range of HCC patients, we first performed an extended bioinformatic analysis of available data from The Cancer Genome Atlas (TCGA). A previous study identified eight genes exhibiting an inverse correlation between DNA methylation (DNAme) and gene expression in HCC patient’s specimens relative to matched normal control, with some of these genes encoding tumor-suppressive proteins [12]. These genes included *CDKN2A*, *CPS1*, *HHIP, MT1E*, *MT1M*, *PTGR1*, *TMEM106A*, and the most abundant hepatic miRNA, *miR-122-5p*. Importantly, integration of copy number variation, gene mutations, and DNAme identified three distinct integrative Clusters (iClust) of HCC patients, being iClust1 patients correlated with one of the poorest outcomes [12].

**Fig. 1:**
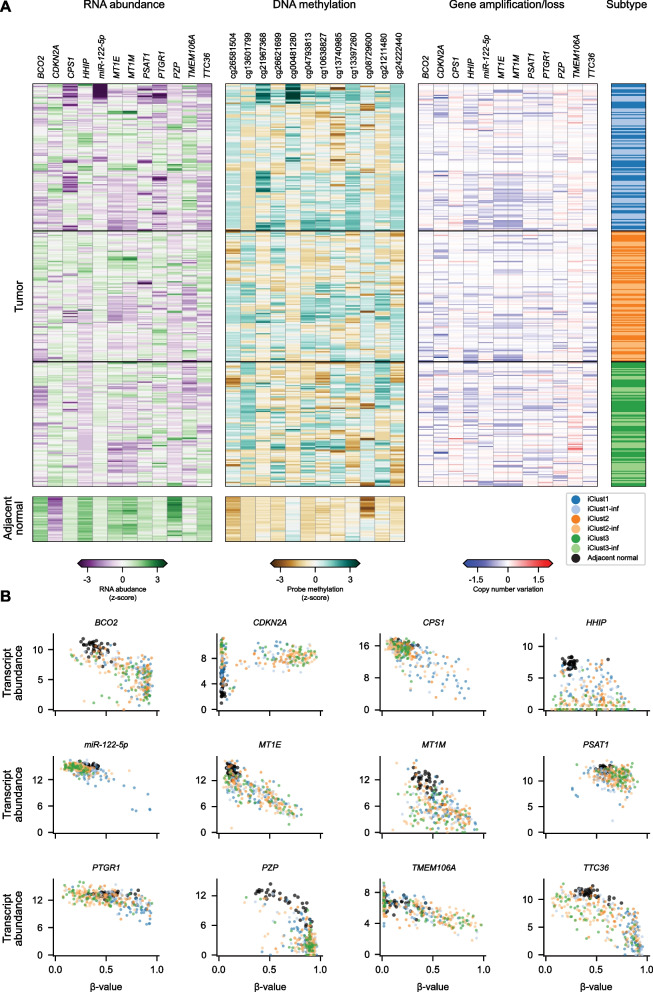
A 12-tumor suppressor gene panel from TCGA hepatocellular carcinoma patients shows gene silencing through promoter DNA methylation within subsets of liver cancer samples. **A** RNA transcript abundance (logTPM, *z*-score normalized), methylation of promoter-associated probes (*β*-value, *z*-score normalized), and copy number variation for the indicated genes (left to right), together with tumor sample “integrated-data cluster” (iClust) annotations. RNA abundance and DNA methylation are also shown for matched normal liver tissue samples where available. **B** Scatter plots showing the association between RNA transcript abundance (*y*-axis; logTPM) and associated probe-level of promoter DNAme (*x*-axis; *β*-value). Scatter markers colors correspond to adjacent normal liver (*black*), iClust1 (*blue*), iClust1-inf (*light blue*), iClust2 (*orange*), iClust2-inf (*light orange*), iClust3 (*green*), or iClust3-inf (*light green*) tumor samples. Further information on inferred iClusters (iClust-inf) is given in “Methods”

To pursue a more comprehensive approach, we utilized computational methods to infer the values of unspecified samples within the three iClusts, leading to the analysis of 357 tumor specimens, subdivided into iClust1 (*n* = 64), iClust1-inf (*n* = 66), iClust2 (*n* = 55), iClust2-inf (*n* = 61), iClust3 (*n* = 62), and iClust3-inf (*n* = 49) (Additional file 1: Fig. S1 and Additional file 12: Data S1). We also searched for additional genes with a strong inverse correlation between RNA abundance and DNAme levels within associated probes, and where the RNA abundance was generally higher within matched normal tissue samples (*n* = 40), where available (Additional file 13: Data S2, Additional file 14: Data S3). This analysis of 397 patients identified four additional candidate TSGs: *BCO2*, *PSAT1*, *PZP*, and *TTC36* (Fig. 1). Each gene captured subsets of patients within previously identified iClusters with evidence of reduced gene expression and promoter DNA hypermethylation relative to adjacent normal liver samples (*black markers*). Genes such as *BCO2* and *PZP* covered relatively large numbers of patients across all three subsets, while *TTC36*, *CPS1*, and *miR-122-5p* showed enrichment within subsets of iClust1 (*blue*), and lastly, *PSAT1* within subsets of iClust2 (*orange*) and iClust3 (*green*) patients.

While no individual gene showed evidence of DNA hypermethylation across all patients (Additional file 2: Fig. S2), and conversely, no individual patient showed promoter DNA hypermethylation across the full 12-gene panel, subsets of patients could be targeted by appropriate combinations, e.g., *BCO2*, *CDKN2A*, *PTGR1*, or *HHIP* for targeting iClust1 tumors; *CDKN2A*, *BCO2*, *PSAT1*, or *MT1E* for either iClust2 or iClust3 tumors; and *CDKN2A*, *PSAT1*, *MT1E*, or *TMEM106A* for iClust3 tumors (Additional file 2: Fig. S2). These data raise the possibility of augmenting the anticancer effect by creating “multiplexed” libraries of TSG reactivation constructs based on CRISPRa technology, thus achieving personalized therapy through tailored combinations of patient biomarkers.

### Epigenetic inhibitors (epi-drugs) reactivate a subset of TSGs in the Hep3B and HuH-7 HCC cell lines

**Fig. 2:**
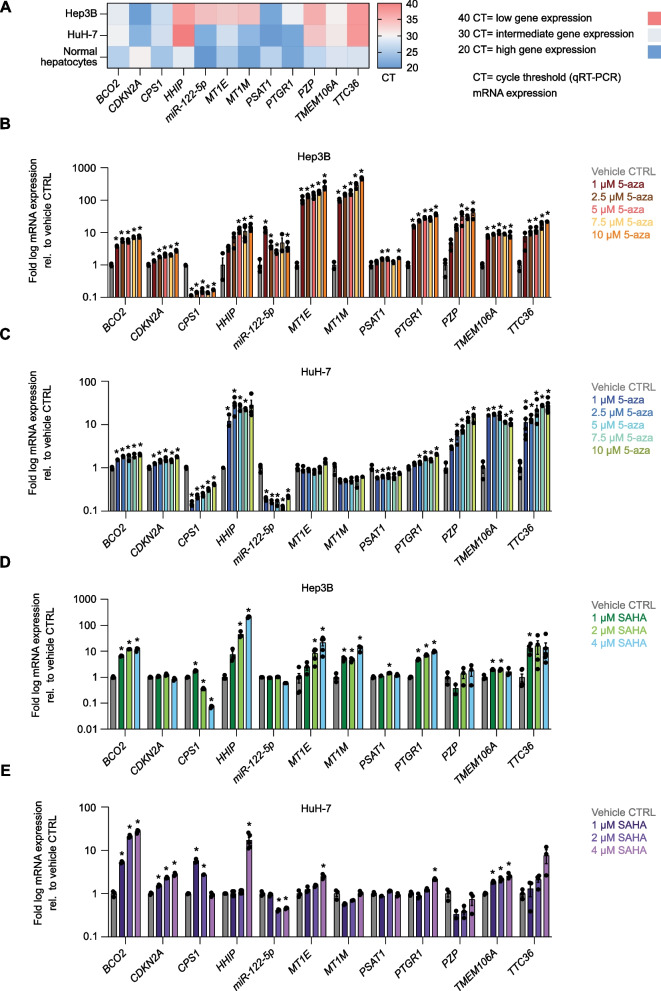
Epi-drugs reactivate a subset of tumor suppressor genes in Hep3B and HuH-7 HCC cell lines.** A** Heatmap of the 12-tumor suppressor gene (TSG) panel comparing the mRNA expression in Hep3B and HuH-7 HCC cell lines versus normal hepatocytes. Gene expression was evaluated by qRT-PCR. Red color indicates the least expressed (cycle threshold 40) and blue color the highest expressed (cycle threshold 20) genes. Data are means (*n* = 3). **B**, **C** Transcriptional regulation of the 12-TSG panel 72 h after 5-aza treatment at different concentrations, indicated by color, in Hep3B and HuH-7 cells, respectively. The data show the fold log10 change in TSG mRNA levels relative to vehicle-treated cells. From left to right, for Hep3B cells: *BCO2*: *P = 0.000017, 0.000189, 0.000237, 0.000041, 0.000036; *CDKN2A*: *P = 0.001726, 0.000067, 0.000965, 0.000064, 0.000066; *CPS1*: *P = 0.000004, 0.000004, 0.000009, 0.000004, 0.000005; *HHIP*: *P = 0.001481, 0.032448, 0.003285; *miR-122-5p*: *P = 0.000884, 0.009364, 0.009338, 0.021386; *MT1E*: *P = 0.013894, 0.000860, 0.008483, 0.001014, 0.021049; *MT1M*: *P = 0.001025, 0.000295, 0.004843, 0.007027, 0.000230; *PSAT1*: *P = 0.011710, 0.020228, 0.004350; *PTGR1*: *P = 0.000021, 0.000004, 0.000007, 0.000130, 0.000010; *PZP*: *P = 0.000595, 0.018607, 0.000256, 0.006314; *TMEM106A*: *P = 0.000067, 0.000002, 0.000175, < 0.000001, 0.002216; and *TTC36*: *P = 0.003576, 0.001630, 0.001968, < 0.000001. For HuH-7 cells: *BCO2*: *P = 0.000234, 0.000074, 0.009780, 0.002345, 0.000073; *CDKN2A*: *P = 0.002474, 0.002569, 0.004410, 0.001787, 0.000041; *CPS1*: *P = 0.000012, 0.000018, 0.000023, 0.000031, 0.000052; *HHIP*: *P = 0.011967, 0.008680, 0.000309, 0.000009; *miR-122-5p*: *P = 0.002266, 0.001959, 0.002015, 0.001671, 0.002592; *PSAT1*: *P = 0.009506, 0.012759, 0.022472, 0.020526; *PTGR1*: *P = 0.008993, 0.000421, 0.002261, 0.000051; *PZP*: *P = 0.000718, 0.012523, 0.000963, 0.000180, 0.001813; *TMEM106A*: *P = 0.000001, 0.000003, 0.000603, 0.000005, 0.000717; and *TTC36*: *P = 0.012646, 0.000005, 0.002686, < 0.000001, 0.000026. **D**, **E** Transcriptional regulation of the 12-TSG panel 48 h after SAHA treatment at different concentrations, indicated by color, in Hep3B and HuH-7 cells, respectively. The data show the fold log10 change in TSG mRNA levels relative to vehicle-treated cells. From left to right, for Hep3B cells: *BCO2*: *P = 0.000006, < 0.000001, 0.000511; *CPS1*: *P = 0.000209, 0.000020, 0.000003; *HHIP*: *P = 0.003836, 0.000015; *MT1E*: *P = 0.004923, 0.005036; *MT1M*: *P = 0.001981, 0.001938, 0.003620; *PSAT1*: *P = 0.000809; *PTGR1*: *P = 0.000052, 0.000013, 0.000023; *TMEM106A*: *P = 0.001558, 0.002973; and *TTC36*: *P = 0.000448. For HuH-7 cells: *BCO2*: *P = 0.000019, 0.000023, 0.000054; *CDKN2A*: *P = 0.000785, 0.000030, 0.000190; *CPS1*: *P = 0.000156, < 0.000001; *HHIP*: *P = 0.005756; *miR-122-5p*: *P = 0.000968, 0.001295; *MT1E*: *P = 0.002001; *PTGR1*: *P = 0.000127; and *TMEM106A*: *P = 0.000221, 0.001073, 0.001910. The data presented as means ± SEM (*n* = 3), and *P* values were determined by multiple unpaired* t*-test comparisons with a two-stage linear step-up procedure of Benjamini, Krieger, and Yekutieli

To validate the functional association between epigenetic regulation and transcriptional downregulation in the context of the 12-TSG gene panel, we investigated the effect of epi-drugs on mutually exclusive epigenetic marks such as DNAme and histone acetylation in representative HCC (Hep3B and HuH-7) cell lines. As observed in HCC clinical specimens, these lines demonstrated downregulation of several TSG’s mRNAs relative to non-transformed hepatocytes, particularly *HHIP*, *miR-122-5p*, *MT1E*, *MT1M*, *PZP*, and *TTC36* in Hep3B, and *HHIP*, *PZP, TMEM106A,* and *TTC36* in HuH-7 cells (Fig. 2A).

We subsequently treated both cell lines with clinically approved epigenetic inhibitors of DNA methyltransferases (DNMTs) and pan-histone deacetylases (HDACs) and monitored TSG expression by quantitative real-time PCR (qRT-PCR). The range of concentrations of 5-aza and SAHA and the duration of the treatments were selected to maximize gene upregulation while minimizing potential toxicities as reported in previous works [25–30]. As expected, pharmacological inhibition of DNMTs by decitabine (5-aza) or HDACs by vorinostat (SAHA) resulted in the reactivation of several TSGs. Treatment with 5-aza led to a significant de-repression of most TSGs in Hep3B cells (Fig. 2B) and, to a lesser extent, in HuH-7 cells (Fig. 2C), in accordance with the basal TSG mRNA abundance (Fig. 2A). This is also consistent with the bioinformatic analyses we performed on the available data, extracted from the Cancer Cell Line Encyclopedia (CCLE), of RNA abundance and DNAme (expressed as *β*-value) for Hep3B and HuH-7 cell lines (Additional file 3: Fig. S3).

In comparison with 5-aza, SAHA led to a lower but still significant degree of TSG mRNA upregulation. Furthermore, treatment with increasing concentrations of epigenetic inhibitors resulted in gene reactivation in a dose-dependent manner only for a subset of TSGs, including *BCO2*, *CDKN2A*, *HHIP*, *MT1E*, *MT1M*, *PTGR1*, and *PZP* (Fig. 2B–E). Unexpectedly, *CPS1* exhibited potent downregulation following treatment with 5-aza in both cell lines (Fig. 2B, C) or with SAHA in Hep3B (Fig. 2D). Similarly, *miR-122-5p* unexpectedly exhibited downregulation upon both treatments in HuH-7 cells (Fig. 2C, E).

Collectively, these data indicate that epi-drugs de-repress a subset of TSGs, albeit non-selectively. Moreover, unexpected significant transcriptional repression was observed for TSGs having basal levels of expression in the aforementioned lines (Fig. 2A), such as *CPS1* and *miR-122-5p*.

### CRISPR/*Sp*dCas9-activation (CRISPRa) systems selectively reactivate TSGs in Hep3B and HuH-7 HCC cell lines

**Fig. 3:**
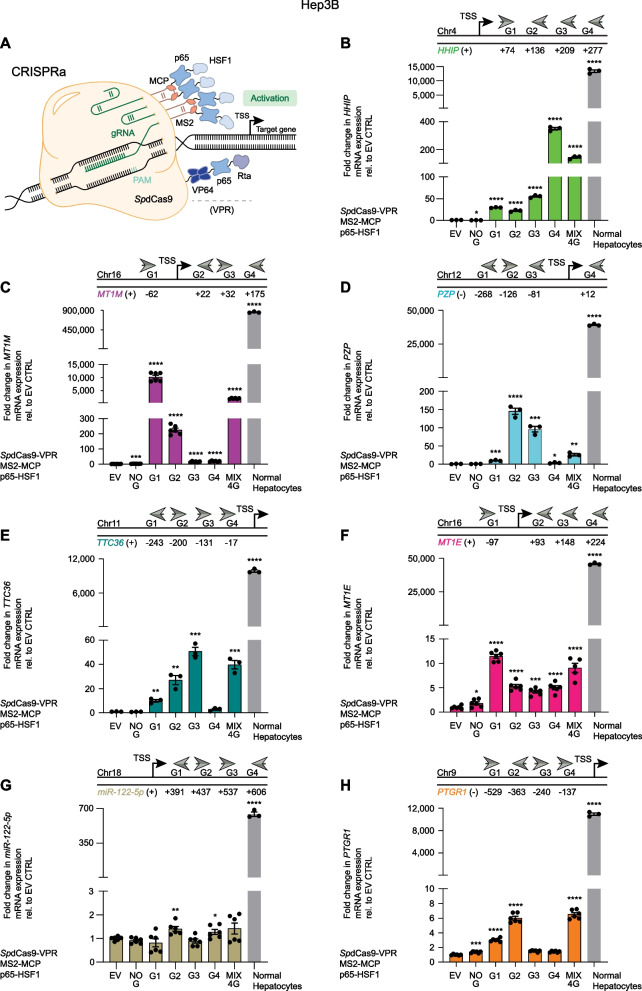
Upregulation of tumor suppressor genes by CRISPRa in Hep3B HCC cells. **A** Schematic representation of CRISPRa consisting of *Sp*dCas9 C-terminally fused to the tripartite transactivator VPR (VP64, p65, and Rta) and coupled with the gRNA-MS2-MCP system that recruits the bipartite transactivator p65-HSF1 for targeted epigenetic editing. As indicated for each tumor suppressor gene (TSG), gRNAs, designated as G1, G2, G3, and G4, direct CRISPRa to the forward (right arrow) or reverse (left arrow) DNA strand within the regulatory region and proximal promoter of the TSG. gRNA numbering (±) refers to the distance in base pairs from the transcription start site (TSS) of each targeted TSG. **B**–**H** Reactivation of TSGs by CRISPRa was evaluated by qRT-PCR 48 h after transient transfection. Fold change in TSG mRNA expression from transfected cells with CRISPRa and TSG-targeting gRNAs, and normal hepatocytes was normalized to control transfections with empty vector (EV) and compared to CRISPRa with no gRNA (NO G) for statistical analysis. From left to right: **B** *HHIP*: *P = 0.0390, ****P < 0.0001; **C** *MT1M*: ***P = 0.0004, ****P < 0.0001; **D** *PZP*: ***P = 0.0006, ****P < 0.0001, ***P = 0.0002, *P = 0.0359, **P = 0.0013, ****P < 0.0001; **E** *TTC36*: **P = 0.0013, **P = 0.0022, ***P = 0.0001, ***P = 0.0004, ****P < 0.0001; **F** *MT1E*: *P = 0.0349, ****P < 0.0001, ***P = 0.0002; **G** *miR-122-5p*: **P = 0.0014, *P = 0.0119, ****P < 0.0001; **H** *PTGR1*: ***P = 0.0001, ****P < 0.0001. Data presented as means ± SEM (*n* = 3), and *P* values were determined by unpaired *t-*test. *Sp*dCas9 *Streptococcus pyogenes* deactivated Cas9 protein adopted for epigenome engineering, MS2 RNA aptamer, MCP MS2 coat protein, HSF1 heat shock factor 1, Chr chromosome, (+) forward DNA strand, (−) reverse DNA strand, and MIX 4G combination of all four gRNAs targeting a TSG

To investigate the focal and selective reactivation of individual TSGs, we exploited CRISPRa technology in a “hit-and-run” (transient transfection) approach. CRISPRa consists of the synergistic combination of *Sp*dCas9, C-terminally fused to the tripartite transcriptional activator domain VPR (VP64, p65, and Rta) [31] with the gRNA-MS2-MCP (MS2-coat protein) which directs genomic specificity (Fig. 3A). This strategy enables the concomitant recruitment of the bipartite p65-HSF1 (heat shock factor 1) activator effector domains (EDs) [32] to the targeted genomic site.

**Fig. 4:**
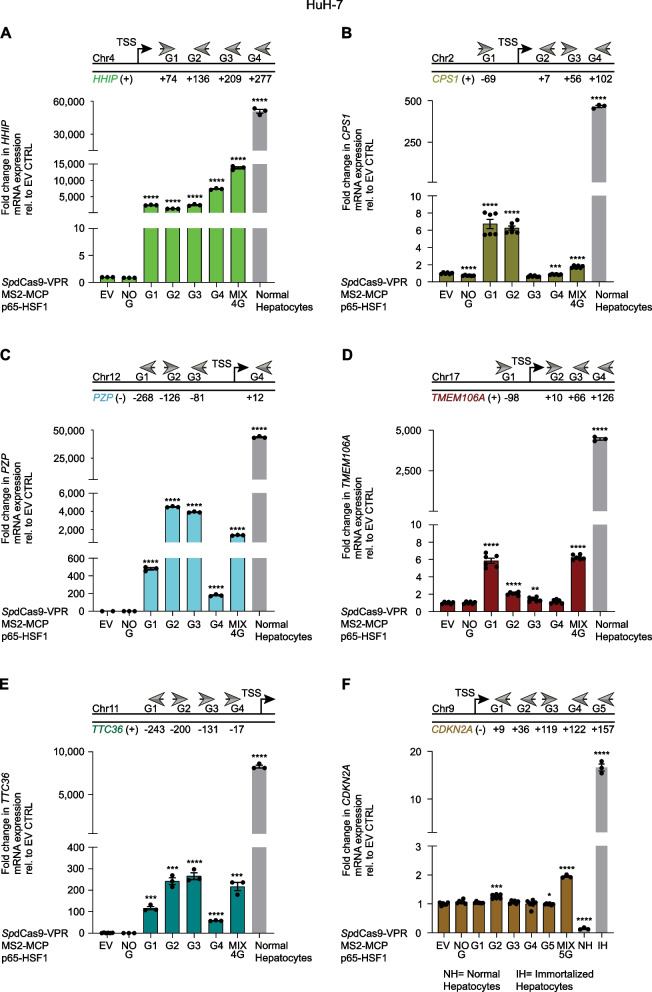
Upregulation of tumor suppressor genes by CRISPRa in HuH-7 HCC cells. **A**–**F** Reactivation of TSGs by CRISPRa was evaluated by qRT-PCR 48 h after transient transfection. Fold change in TSG mRNA expression from transfected cells with CRISPRa and TSG-targeting gRNAs, normal and immortalized hepatocytes was normalized to control transfections with empty vector (EV), and compared to CRISPRa with no gRNA (NO G) for statistical analysis. From left to right: **A** *HHIP*: ****P < 0.0001; **B** *CPS1*: ****P < 0.0001, ***P = 0.0005; **C** *PZP*: ****P < 0.0001; **D** *TMEM106A*: ****P < 0.0001, **P = 0.0063; **E** *TTC36*: ***P = 0.0001, ****P < 0.0001, ***P = 0.0003; **F** *CDKN2A*: ***P = 0.0010, *P = 0.0185, ****P < 0.0001. Data presented as means ± SEM (*n* = 3), and *P* values were determined by unpaired *t-*test

For each TSG we designed four gRNAs targeting the proximal promoter and the nearby regulatory region mapping upstream and downstream of the transcription start site (TSS) (Additional file 10: Table S1). We focused on several candidate TSGs for each cell line having low or intermediate levels of basal expression relative to normal hepatocytes (Fig. 2A): *HHIP*, *MT1M*, *PZP*, *TTC36, MT1E, miR-122-5p*, and *PTGR1* in Hep3B cells (Fig. 3) and *HHIP*, *CPS1*, *PZP*, *TMEM106A*, *TTC36*, and *CDKN2A* in HuH-7 cells (Fig. 4). The gRNAs were selected based on established algorithms that predict both maximal on- and minimal off-target binding sites [33, 34] and qRT-PCR was performed 48 h post-transfection to quantitate mRNA expression; normal (non-transformed) human hepatocyte mRNA was processed as a reference control.

We observed significant CRISPRa-mediated transcriptional upregulation of all the TSGs targeted relative to the empty vector control (EV) or to CRISPRa in absence of gRNA (NO G). However, the activation of TSGs achieved by CRISPRa in HCC lines partially reached the physiological mRNA levels expressed in normal hepatocytes (Figs. 3, 4). Notably, one specific gRNA was sufficient to attain the strongest gene reactivation for most of the TSGs examined, as in the case for *HHIP* (gRNA 4 (G4) vs. EV, 349.5-fold, *P* < 0.0001), *MT1M* (G1, 10,211-fold, *P* < 0.0001), *PZP* (G2, 145.3-fold, *P* < 0.0001), *TTC36* (G3, 50.6-fold, *P* = 0.0001), *MT1E* (G1, 11.45-fold, *P* < 0.0001), *miR-122-5p* (G2, 1.4-fold, *P* = 0.0014), and *PTGR1* (G2, 6.0-fold, *P* < 0.0001) in Hep3B cells (Fig. 3B–H). A similar pattern was observed in HuH-7 cells for *PZP* (G2 vs. EV, 4,500-fold, *P* < 0.0001), *TTC36* (G3, 265.6-fold, *P* < 0.0001), *CPS1* (G1, 6.7-fold, *P* < 0.0001), and *TMEM106A* (G1, 5.8-fold, *P* < 0.0001, nearly as much as the MIX 4G, 6.2-fold, *P* < 0.0001) (Fig. 4B–E). For *HHIP* and *CDKN2A* in HuH-7 cells, the combinations of all designed gRNAs (MIX) were necessary to maximize TSG activation, i.e., the mix of four guides (MIX 4G vs. EV, 13,852-fold, *P* < 0.0001) (Fig. 4A), and the mix of five guides (MIX 5G, 1.95-fold, *P* < 0.0001) (Fig. 4F), respectively. Importantly, CRISPRa enabled all the targeted TSGs to be upregulated relative to EV or NO G controls, despite their basal gene expression, in contrast to the epi-drugs, which led to unexpected downregulation of some TSGs, such as *CPS1* and *miR-122-5p*.

Moreover, CRISPRa outperformed the epi-drug treatments (5-aza or SAHA, all concentrations tested) in upregulating *HHIP*, *MT1M*, *PZP*, *TTC36*, and *CPS1*, whereas 5-aza, but not SAHA led to a higher activation of *MT1E*, *miR-122-5p*, *PTGR1*, and *TMEM106A* compared to CRISPRa. Only the highest doses of SAHA (2 and 4 µM) exceeded CRISPRa-based upregulation of *PTGR1* and *CDKN2A* (Additional file 15: Data S4).

To investigate unintended gene modulation by CRISPRa, we bioinformatically analyzed the most active gRNA sequences, for each of the ten targeted TSGs, for genome-wide mismatches equal to or less than three mispairs [35]. We, then, matched the genomic location of the putative gRNA off-target binding sites in the human genome browser to identify those in the proximity of genomic regulatory elements, i.e., promoters and enhancers. Next, qRT-PCR was conducted on the potential off-targets to assess their transcriptional regulation by CRISPRa (Additional file 4, 5: Fig. S4 and Additional file 16: Data S5). We found no significant gene modulation of any of the bioinformatically predicted (potential) off-target genes, consistently with previous works [36], suggesting negligible off-target activities.

### *Sp*dCas9-VPR and MS2-MCP-p65-HSF1 is the most potent CRISPRa platform for “hit-and-run” TSG reactivation

**Fig. 5:**
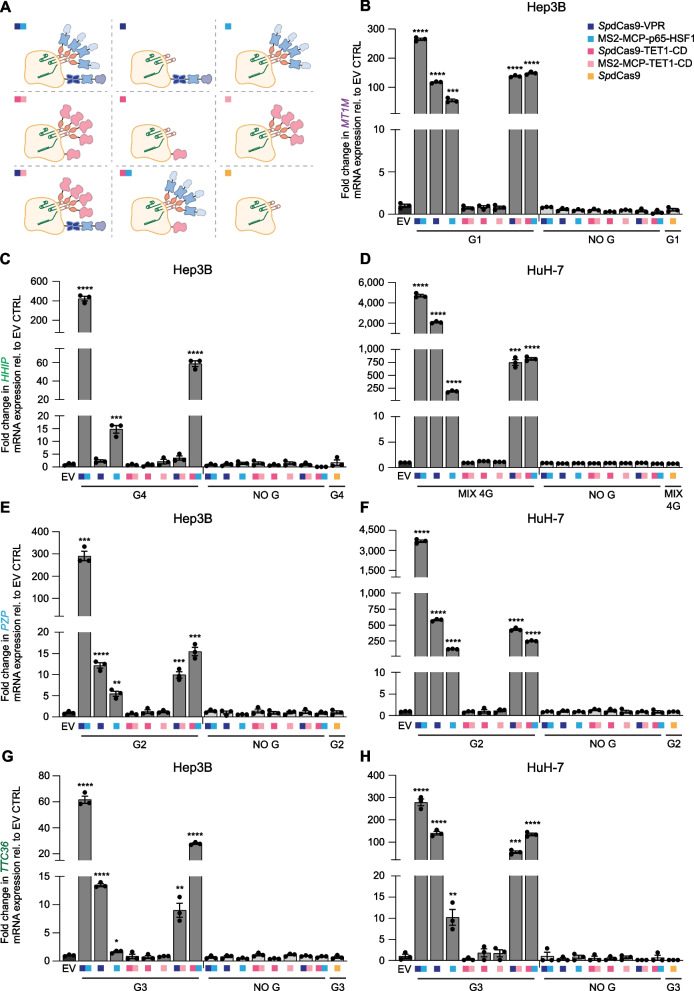
Maximizing reactivation of highly downregulated tumor suppressor genes in Hep3B and HuH-7 HCC cells.** A** Schematic representation depicting the CRISPRa toolbox developed for epigenetic editing. *Sp*dCas9 C-terminally fused to VPR, i.e., *Sp*dCas9-VPR (*dark blue*); gRNA-MS2-MCP-p65-HSF1 recruiting system (*light blue*); *Sp*dCas9 C-terminally fused to TET1 catalytic domain, i.e., *Sp*dCas9-TET1-CD (*dark pink*); gRNA-MS2-MCP-TET1-CD recruiting system (*light pink*); and *Sp*dCas9 (*yellow*). **B**–**H** Fold change in *MT1M* (**B**), *HHIP* (**C**, **D**), *PZP* (**E**, **F**), and *TTC36* (**G**, **H**) mRNA expression evaluated by qRT-PCR 96 h after transient transfection in Hep3B and HuH-7 cells. Cells were transfected with combinations of CRISPRa along with the most potent tumor suppressor gene-targeting gRNA/s, or with no gRNA (NO G) as control. Relative gene expression was normalized and compared to cells transfected with empty vector control (EV) for statistical analysis. From left to right: **B** *MT1M*: ****P < 0.0001, ***P = 0.0002; **C** *HHIP*: **** P < 0.0001, ***P = 0.0008; **D** *HHIP*: ****P < 0.0001, ***P = 0.0002; **E** *PZP*: ***P = 0.0002, ****P < 0.0001, **P = 0.0022, ***P = 0.0003, ***P = 0.0001; **F** *PZP*: ****P < 0.0001; **G** *TTC36*: ****P < 0.0001, *P = 0.0190, **P = 0.0030; **H** *TTC36*: ****P < 0.0001, **P = 0.0088, ***P = 0.0007. Data presented as means ± SEM (*n* = 3), and *P* values were determined by unpaired *t-*test. *Sp*dCas9 *Streptococcus pyogenes* deactivated Cas9 protein adopted for epigenome engineering, VPR VP64, p65, Rta, MS2 RNA aptamer, MCP MS2-coat protein, HSF1 heat shock factor 1, TET1-CD Ten-Eleven Translocation methylcytosine dioxygenase 1-catalytic domain, MIX 4G combination of four gRNAs

Since our most downregulated TSGs were marked by DNAme (Additional file 3: Fig. S3), we next investigated whether the DNA demethylase catalytic domain (CD) of TET1, Ten-Eleven Translocation methylcytosine dioxygenase 1, could be exploited to reactivate these genes. We chose *HHIP*, *PZP*, and *TTC36* in Hep3B and HuH-7 cells as well as *MT1M* in Hep3B cells, since these targets had the lowest transcript levels (Fig. 2A), resulting in a strong re-expression upon epi-drug treatment, particularly with 5-aza (Fig. 2B, C), and also given the strong negative correlation between their RNA abundance and DNAme status observed in patients (Fig. 1) as well as in Hep3B and HuH-7 cell lines (Additional file 3: Fig. S3). Furthermore, to identify additional (potentially synergistic) combinatorial strategies for TSG reactivation, we combined TET1-CD and MS2-MCP-TET1-CD with the epigenetic editors VPR and MS2-MCP-p65-HSF1 comprising a compact “epi-toolbox” of eight different CRISPRa (Fig. 5A). Unexpectedly, TET1-CD alone, either tethered to *Sp*dCas9, or recruited by the MS2-MCP system, or even concomitantly fused and recruited, was unable to de-repress any of the TSGs targeted in either of the two HCC lines transiently transfected along with the most potent gRNA/s or with no gRNA as control (Fig. 5B–H). Similarly, there were no changes in *MT1M* nor in *TTC36* mRNA expression when tiling their respective gene promoters by a mix of four gRNAs directing *Sp*dCas9-TET1-CD, or *Sp*dCas9-TET1-CD in combination with MS2-MCP-TET1-CD in Hep3B cells (Additional file 6: Fig. S5). Instead, this combinatorial approach underscored the significant superiority of *Sp*dCas9-VPR synergistically combined with the MS2-MCP-p65-HSF1 system in reactivating all four highly downregulated targeted TSGs (*HHIP*, *MT1M*, *PZP*, and *TTC36*) in the respective cell lines (Fig. 5B–H). A synergistic booster effect was obtained when the tripartite (VPR) and the bipartite (p65-HSF1) activators were co-delivered.

Interestingly, the combinations consisting of *Sp*dCas9-VPR with MS2-MCP-TET1-CD, and *Sp*dCas9-TET1-CD with MS2-MCP-p65-HSF1, also exhibited a significant gene reactivation of all four targeted TSGs, apart from *HHIP* in Hep3B cells targeted by the former (Fig. 5B–H).

To ascertain the functional association between DNAme and the observed *HHIP* re-expression (150-fold, *P* < 0.0001) achieved by *Sp*dCas9-TET1-CD, MS2-MCP-p65-HSF1, and gRNA G4, we performed DNAme analyses (Infinium Methylation EPIC 850K arrays) in Hep3B cells transiently transfected for 96 h (Additional file 7: Fig. S6). Surprisingly, we found no significant targeted DNA demethylation in the *HHIP* proximal promoter, nor for the most potent CRISPRa (*Sp*dCas9-VPR and MS2-MCP-p65-HSF1) guided by the same gRNA G4, which does not engage TET1-CD, but that nevertheless led to the highest *HHIP* re-expression (781-fold, *P* < 0.0001) (Additional file 7: Fig. S6 and Additional file 17: Data S6). Furthermore, no significant genome-wide changes in DNAme were detected for these two CRISPRa transfected conditions, relative to NO G controls.

Since TET1-CD, either fused to *Sp*dCas9 and/or recruited by the gRNA-MS2-MCP-system, did not lead to significant TSG reactivation in transiently transfected cells, we next investigated an “all-in-one” lentiviral vector which enabled the generation of stable cell lines constitutively co-expressing the gRNA along with *Sp*dCas9-TET1-CD. When the *Sp*dCas9-TET1-CD construct was co-expressed with a mix of four gRNAs targeting *HHIP* in Hep3B cells, no significant upregulation was observed relative to NO G control, even 40 days after transduction of the cells, nor in the context of *MT1M* targeted by four single gRNAs (G1, G2, G3, and G4) or their mix, 20 days post-transduction. In contrast, *Sp*dCas9-VPR led to a robust *MT1M* gene de-repression in Hep3B cells (G1 vs. NO G, 17,382-fold, *P* < 0.0001) (Additional file 8: Fig. S7).

Overall, these results suggest that *Sp*dCas9-VPR synergistically combined with the MS2-MCP-p65-HSF1 recruiting system, or the *Sp*dCas9-VPR alone are the most effective CRISPRa platforms for the reactivation of several highly silenced TSGs in HCC cells.

### CRISPRa reactivates TSGs more specifically and more potently than epi-drugs and enables genome multiplexing “at will”

**Fig. 6:**
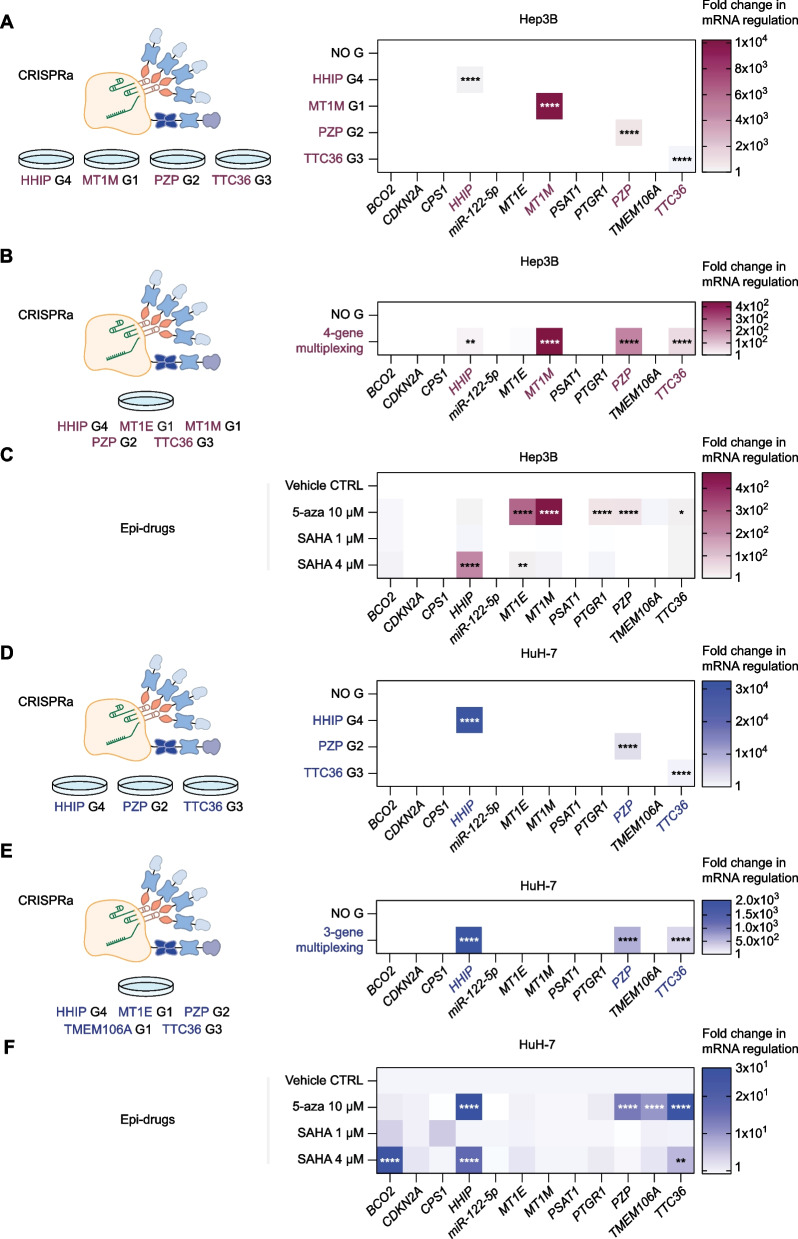
Specific and potent reactivation of silenced tumor suppressor genes by CRISPRa compared to epi-drugs, and CRISPRa-enabled genome multiplexing in Hep3B and HuH-7 HCC cells. **A**–**F** Heatmap of the 12-tumor suppressor gene (TSG) panel comparing the fold change in mRNA regulation evaluated by qRT-PCR 48 h after transient transfections (**A**), (**B**), (**D**) and (**E**), 72 h after 5-aza, or 48 h after SAHA treatments (**C**) and (**F**). The transfection conditions and the epi-drug treatments are arranged in rows, and the genes in columns. Hep3B and HuH-7 cells were either transfected with CRISPRa (*Sp*dCas9-VPR and MS2-MCP-p65-HSF1) along with the most potent TSG-targeting gRNA, or with no gRNA (NO G) as control; or treated with epi-drugs or vehicle control. Relative gene expression was normalized to cells transfected with empty vector control and compared to CRISPRa with NO G (**A**), (**B**), (**D**), and (**E**), or normalized and compared to cells treated with vehicle control (**C**) and (**F**) for statistical analysis. Data presented as means (*n* = 3). **A** Targeted transcriptional regulation of four TSGs by CRISPRa in Hep3B cells. *P* values were determined by two-way ANOVA with Dunnett's multiple comparisons test (HHIP G4: ****P < 0.0001; MT1M G1: ****P < 0.0001; PZP G2: ****P < 0.0001; and TTC36 G3: **** P < 0.0001). **B** Targeted genome multiplexing for simultaneous transcriptional reactivation of four TSGs by CRISPRa in Hep3B cells. *P* values were determined by two-way ANOVA with Šídák's multiple comparisons test (*HHIP*: **P = 0.0092; *MT1E*: *P* = not significant; *MT1M*: ****P < 0.0001; *PZP*: ****P < 0.0001; and *TTC36*: ****P < 0.0001). **C** Untargeted transcriptional regulation of several TSGs in Hep3B cells treated with 10 µM 5-aza (*MT1E*: ****P < 0.0001; *MT1M*: ****P < 0.0001; *PTGR1*: ****P < 0.0001; *PZP*: ****P < 0.0001; and *TTC36*: *P = 0.0207), 1 µM SAHA, or 4 µM SAHA (*HHIP*: ****P < 0.0001; and *MT1E*: **P = 0.0089). *P* values were determined by two-way ANOVA with Dunnett's multiple comparisons test. **D** Targeted transcriptional regulation of three TSGs by CRISPRa in HuH-7 cells. *P* values were determined by two-way ANOVA with Šídák's multiple comparisons test. (HHIP G4: ****P < 0.0001; PZP G2: ****P < 0.0001; and TTC36 G3: ****P < 0.0001). **E** Targeted genome multiplexing for simultaneous transcriptional reactivation of three TSGs by CRISPRa in HuH-7 cells. *P* values were determined by two-way ANOVA with Šídák's multiple comparisons test (*HHIP*: ****P < 0.0001; *MT1E*: *P* = not significant; *PZP*: ****P < 0.0001; *TMEM106A*: *P* = not significant; and *TTC36*: ****P < 0.0001). **F** Untargeted transcriptional regulation of several TSGs in HuH-7 cells treated with 10 µM 5-aza (*HHIP*: ****P < 0.0001; *PZP*: ****P < 0.0001; *TMEM106A*: ****P < 0.0001; and *TTC36*: ****P < 0.0001), 1 µM SAHA, or 4 µM SAHA (*BCO2*: ****P < 0.0001; *HHIP*: ****P < 0.0001; and *TTC36*: **P = 0.0072). *P* values were determined by two-way ANOVA with Dunnett's multiple comparisons test

We next compared the specificity and the potency of CRISPRa technologies versus traditional epi-drug-based inhibition (decitabine and vorinostat) for the reactivation of TSGs in "hit-and-run” approaches (transient transfection of CRISPR vectors *Sp*dCas9-VPR and MS2-MCP-p65-HSF1). To this aim, we focused on the most downregulated TSGs (*HHIP*, *MT1M*, *PZP*, and *TTC36*) in Hep3B (Fig. 6A–C) and *HHIP*, *PZP*, and *TTC36* in HuH-7 (Fig. 6D–F). Cells were either transfected with an optimized CRISPRa (*Sp*dCas9-VPR and MS2-MCP-p65-HSF1) platform and single gRNA targeting either single TSGs, or by combining multiple gRNAs to assess the efficacy of TSG multiplexing. Fold-transcriptional changes were cross-examined by qRT-PCR, and data were normalized and visualized in heatmap plots relative to NO G control. In contrast to 5-aza and SAHA, which caused uncontrolled concurrent reactivation of several TSGs relative to vehicle control, CRISPRa platforms demonstrated a high degree of on-target specificity in both cell lines, with non-detectable off-target effects. Notably, the degree of non-specific TSG reactivation induced by the epi-drugs depended on the nature of the inhibitor used (with 5-aza being stronger than SAHA) and varied upon the concentrations utilized. Additionally, by comparing the fold-change in cognate mRNA regulation achieved between CRISPRa technology (Fig. 6A, D) with those of the epi-drug treatments (Fig. 6C, F), we observed higher activation of CRISPRa systems by several orders of magnitude relative to the conventional inhibitors in de-repressing individual TSGs. The fold changes obtained were dependent on the type of the epi-drug and the concentrations utilized, as well as the target gene, and it is cell-type specific (Additional file 15: Data S4). Importantly, even when performing up to 4-TSG multiplexing, the fold changes achieved in TSG mRNA regulation employing CRISPRa were significantly higher than those observed with the epi-drugs (Fig. 6B–C, E–F).

Taken together, these data demonstrate that CRISPRa enabled at command “genome multiplexing,” facilitating the simultaneous reactivation of up to four TSGs, and reaching significantly higher levels of activation than those obtained with the epi-drugs for most of the TSGs targeted (Additional file 15: Data S4).

### Reactivation of TSGs, either individually or in combination, via CRISPRa, reduces HCC cell proliferation, cell viability, and cell migration

**Fig. 7:**
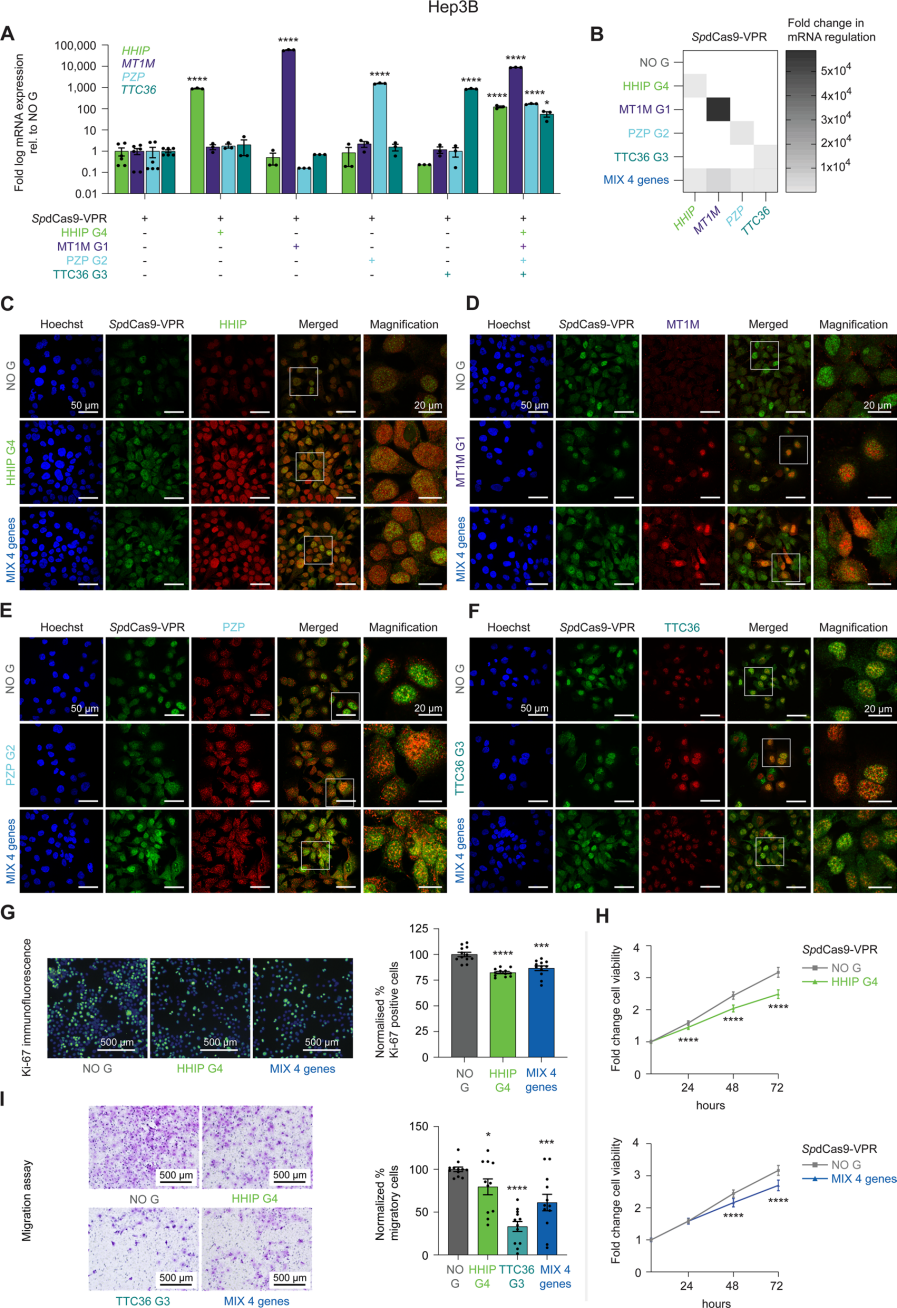
Reactivation of silenced tumor suppressor genes by CRISPRa correlates with phenotypic reprogramming in Hep3B HCC cells. **A**, **B** Transcriptional reactivation of four silenced tumor suppressor genes (TSGs), *HHIP*, *MT1M*, *PZP*, and *TTC36,* targeted individually and simultaneously (MIX 4 genes) by *Sp*dCas9-VPR stably expressed along with the corresponding TSG-gRNA in Hep3B HCC cells.** A** Data shown as fold log10 change in TSG mRNA levels, evaluated by qRT-PCR, relative to *Sp*dCas9-VPR with no gRNA (NO G). Data presented as means ± SEM (*n* = 3) and *P* values were determined by one-way ANOVA with Dunnett's multiple comparisons test (****P < 0.0001, *P = 0.0107). **B** Heatmap comparing the fold change in mRNA regulation evaluated by qRT-PCR. The TSG-gRNAs are arranged in rows, and the genes in columns. Data presented as means (*n* = 3). **C**–**F** Immunofluorescence of HHIP, MT1M, PZP, TTC36, *Sp*dCas9-VPR, and Hoechst-stained cell-nuclei in stable Hep3B cells expressing *Sp*dCas9-VPR alone (NO G), *Sp*dCas9-VPR targeting HHIP with G4, MT1M with G1, PZP with G2, TTC36 with G3, or *Sp*dCas9-VPR co-targeting all four TSGs (MIX 4 genes). **G**–**I** Phenotypic reprogramming in Hep3B cells lentivirally transduced with *Sp*dCas9-VPR targeting and upregulating *HHIP* with G4, *TTC36* with G3, the MIX 4 genes, or with NO G as control. **G** Cell proliferation assessed by α-Ki-67 immunostaining (*green*), superimposed on nuclear Hoechst 33258 staining (*blue*). Data normalized to *Sp*dCas9-VPR NO G, presented as means ± SEM (*n* = 3), and *P* values were determined by unpaired *t-*test (****P < 0.0001, ***P = 0.0003). **H** Cell viability determined using a luminescence assay (CellTiter-Glo^®^). Data shown as fold change compared to *Sp*dCas9-VPR NO G at 24, 48, and 72 h, presented as means ± SEM (*n* = 3), and *P* values were determined by unpaired *t-*test with Welch's correction (****P < 0.0001). **I** Inhibition of cell migration assessed by the Boyden chamber assay. Data normalized to *Sp*dCas9-VPR NO G, presented as means ± SEM (*n* = 3), and *P* values were determined by unpaired *t-*test (*P = 0.0390, ****P < 0.0001, ***P = 0.0008)

We next investigated whether the reactivation of TSGs targeted individually and/or multiplexed by CRISPRa technology led to associated phenotypic cell reprogramming of HCC cells. To bypass the high cell-to-cell phenotypic variability reported in transient transfection assays due to a large number of plasmids transfected as well as the heterogeneous sizes (gRNA vs. *Sp*dCas9-VPR) of the plasmids, we generated stable, lentivirally transduced, Hep3B cell lines. These transduced cells constitutively expressed the “all-in-one” lentiviral vector encoding *Sp*dCas9-VPR and the most potent gRNA for each targeted TSG (Fig. 7). We focused on the four highly silenced TSGs: *HHIP*, *MT1M*, *PZP*, and *TTC36* in Hep3B cells. As shown in Fig. 7A, B, we achieved potent TSG re-expression by targeting the four TSGs, either individually (*HHIP*, G4 vs. NO G, 883-fold, *P* < 0.0001; *MT1M*, G1, 57,534-fold, *P* < 0.0001; *PZP*, G2, 1,541-fold, *P* < 0.0001; and *TTC36*, G3, 856-fold, *P* < 0.0001), or as 4-gene multiplexing (MIX 4 genes) by simultaneously co-delivering multiple gRNAs (*HHIP*, G4 vs. NO G, 121-fold, *P* < 0.0001; *MT1M*, G1, 8,745-fold, *P* < 0.0001; *PZP*, G2, 167-fold, *P* < 0.0001; and *TTC36*, G3, 55-fold, *P* = 0.0107). Similar to transient systems, we observed no significant cross-gene modulation within the four targeted TSGs (Fig. 7B) nor in the bioinformatically identified potential off-target genes (Additional file 4, 5: Fig. S4), outlining the high specificity of CRISPRa technology, even with constitutively expressed lentiviral vectors.

Immunofluorescence assays of the transduced cells demonstrated that *Sp*dCas9-VPR was expressed mainly in the nucleus of the Hep3B cells, whereas HHIP was detected in the nucleus and plasma membrane. The MT1M and TTC36 targets showed nuclear localization, and PZP was predominantly found in the extracellular space and plasma membrane as well as in the cytoplasm (perinuclear). These data show that the TSG mRNA upregulation achieved by CRISPRa was sufficient to elicit changes in expression at protein level, even for targets that were mostly silenced in control cells with undetectable protein levels, such as *MT1M*. Importantly, all these 4 co-targeted TSGs were found upregulated in the multiplexing (MIX 4 genes) condition, outlining the efficiency of CRISPRa in reactivating multiple targets at once (Fig. 7C–F).

To correlate the extent of TSG re-expression with cancer cell phenotypic remodeling, Hep3B cells transduced with *Sp*dCas9-VPR targeting *HHIP* or the multiplexed four TSGs (*HHIP*, *MT1M*, *PZP*, and *TTC36)* were processed by cell proliferation, cell viability, and cell migration assays. We observed a significant decrease in the expression of the proliferative marker Ki-67 by 18% for HHIP G4 (*P* < 0.0001) and by 13% for MIX 4 genes (*P* = 0.0003), compared to *Sp*dCas9-VPR NO G (Fig. 7G). Similarly, the impact on tumor cell viability over 72 h was found more pronounced upon targeting HHIP with G4 alone (*P* < 0.0001), although still significantly lessened in the MIX 4 genes condition (*P* < 0.0001) relative to NO G control (Fig. 7H). These analyses demonstrated that HHIP was a key TSG controlling liver cancer cell proliferation and cell viability; in fact, only modest changes in cell viability were achieved when individually targeting *MT1M*, *PZP*, and *TTC36* (Additional file 9: Fig. S8).

Interestingly, there was a significant reduction (77%, ~ 40%, and 20%) in migratory cells in the TTC36 G3, in the MIX 4 genes, and in the HHIP G4 conditions, respectively, vs. NO G control (*P* < 0.0001, *P* = 0.0008, *P* = 0.0390) (Fig. 7I), whereas neither MT1M G1 nor PZP G2 conditions perturbed the migratory capacity in the respective stable Hep3B cell lines (Additional file 9: Fig. S8).

In conclusion, these data demonstrate that CRISPRa is a potent and highly specific platform to interrogate the functional role of silenced TSGs. We also demonstrate that multiplexing of various TSGs facilitates the functional regulation of various facets of TSG biology at once, in this case, regulation of cell growth and migration, making it a promising precision oncology tool for the treatment of liver cancer.

## Discussion

We have developed a CRISPRa toolbox of epigenetic effector domains and gRNAs for the targeting of single and multiple combinations of TSGs in HCC cells. This approach could be harnessed for the functional interrogation of TSGs, and, ultimately, as a precision medicine for the treatment of aggressive forms of HCCs. These patients have few treatment options, and their clinical benefit is limited, particularly for late-stage HCC, as they often develop resistance.

Our computational analysis validated a 12-gene TSG panel which comprehensively captures all available HCC patients across the three main integrative clusters (iClusts) of HCC. These TSGs are both downregulated at the mRNA level and marked by DNA promoter methylation in clinical cohorts of HCC patients. We demonstrated that the CRISPRa approach represents a versatile toolkit to multiplex TSGs with high precision, potentially representing innovative tailored treatments for the different subtypes of HCC.

Our 12-gene TSG panel comprises: (1) regulators of cell cycle, e.g., *CDKN2A* [13, 14]; (2) metabolic enzymes such as *BCO2* [37, 38] (a regulator of lycopene metabolism), *CPS1* [39] (a liver-specific, intramitochondrial, rate-limiting enzyme in the urea cycle), *PTGR1* [40] (a bifunctional enzyme that inactivates leukotrienes and prostaglandins), and *PSAT1* [41, 42] (a phosphoserine aminotransferase); (3) regulators of stemness, such as *HHIP* [43–49]*,* which is a suppressor of the Hedgehog signaling pathway involved in embryonic development and tumorigenicity; (4) pro-apoptotic factors including the metallothioneins *MT1E* [50, 51] and *MT1M* [52–54]*,* which act as a surveillance systems for carcinogens-caused cellular damage; immune-regulators, such as the transmembrane protein *TMEM106A* [55, 56] (an activator of the MAPK and NF-κB signaling pathways implicated in the pro-inflammatory and anti-tumoral M1-type macrophage polarization); and (5) negative regulators of migration, invasion, angiogenesis, and metastasis, such as the liver specific *miR-122-5p* [57, 58] (a post-transcriptional regulator of genes involved in TNF and Notch signaling pathways), *PZP* [59–61] (a proteinase inhibitor), and *TTC36* [62–64] (a regulator of the Wnt-*β*-catenin pathway). Thus, while we focused on a limited array of phenotypic assays, the multiplexing approach has great potential to reprogram multiple aspects of HCC pathobiology, which could potentially facilitate a more comprehensive and multi-factorial reprogramming of the HCC phenotype, including the tumor microenvironment.

To activate the array of 12 TSGs, we investigated CRISPRa platforms combining different gRNAs and effector domains. In contrast to epigenetic drugs (decitabine and vorinostat) that led to a non-selective transcriptional modulation, we showed that CRISPRa systems enabled specific reactivation of single and multiple TSGs with negligible off-target effects, and were more potent in upregulating the majority of the TSGs targeted in the conditions tested in this study. The most silenced TSGs, including *HHIP*, *MT1M*, *PZP*, and *TTC36* were consistently reactivated by *Sp*dCas9-VPR and MS2-MCP-p65-HSF1 epigenetic editing tools in both HCC cell lines tested in a hit-and-run (transient transfection) approach. This is consistent with our previous study on the TSGs *MASPIN* and *RPRM* in breast and gastric cancer cells [18].

While we did not observe reactivation in any of the four strongly silenced TSGs targeted by employing a DNA demethylase catalytic domain (TET1-CD), neither fused to *Sp*dCas9 and/nor recruited by the gRNA-MS2-MCP system, significant TSG reactivation was achieved when the TET1-CD was combined with other effectors, such as VPR or p65-HSF1. Similarly, *Sp*dCas9-TET1-CD did not elicit significant TSG re-expression in the context of lentivirally transduced cells, neither by four individual gRNAs targeting *MT1M* nor by tiling *MT1M* or *HHIP* promoters with the mixture of four gRNAs. These findings are in contrast to previous works showing that TET1-CD fused to *Sp*dCas9 and/or recruited by the gRNA-MS2-MCP system enabled re-expression of TSGs, such as *BRCA1* in breast and cervical cell lines [65]; *SARI* in colon cancer [66]; *RANKL* [67] in neuroblastoma; several hypermethylated genes in lung adenocarcinoma, via the SunTag platform recruiting TET1-CD and VP64 [68]; and *FMR1* in induced pluripotent stem cells derived from fragile X syndrome patients by *Sp*dCas9-TET1-CD encoded in a lentiviral vector [69]. Moreover, pioneering studies employing retroviral transduction of Zinc-finger proteins engineered with TET-2-CD have shown reactivation of candidate TSGs, such as *C13ORF18* and *TFPI2* in cervical cancer cells [70], and *ICAM-1* in ovarian cancer cells [71].

Finally, we did not observe DNA demethylation changes upon *HHIP* reactivation in any of the CRISPRa epigenetic tools employed, such as *Sp*dCas9-VPR and MS2-MCP-p65-HSF1 or our newly *Sp*dCas9-TET1-CD and MS2-MCP-p65-HSF1 combination in transiently transfected Hep3B cells.

It is worth noting that CRISPRa elicited a partial reactivation of the silenced TSGs targeted compared to their physiological gene expression levels in normal hepatocytes. Nonetheless, this approach was still sufficient to elicit significant phenotypic changes in terms of reduced cell proliferation, cell viability, and cell migration, supporting previous work in the context of other TSGs using zinc finger technologies in cancer [18, 72, 73].

For the majority of the TSGs investigated, a single gRNA was sufficient to drive potent re-expression, although at levels that were lower relative to those of normal hepatocytes. For a small number of TSGs (*HHIP* and *CDKN2A*) in HuH-7 cells, a mix of four or five gRNAs was required to elicit significant gene reactivation. This is also consistent with other studies where tiling the gene promoter led to a higher transcriptional reactivation than that achieved by a single gRNA [74, 75]. Consistent with other reports, our data show that the CRISPRa-gRNA design as well as the specific repertoire of epigenetic modifiers that maximize the gene activation state depends on the chromatin context, the targeted gene, and it is, therefore, cell-type specific [68, 76].

In our hands, the synergistic combination of mechanistically distinct effector domains (EDs), such as VPR and MS2-MCP-p65-HSF1, is a powerful tool to endogenously reactivate silenced TSGs in HCC cell lines. These data are consistent with our previous studies in lung and gastric cancer cells both in vitro [18] and in an in vivo breast cancer model [21]. The combination of mechanistically distinct EDs has the potential to recruit a “cloud” of chromatin modifiers that cooperate to catalyze euchromatin formation in the targeted promoter. This suggests that prudent screening of the available epigenetic editing tools might be required for the maximization of the epigenome engineering in HCC.

Importantly, our functional assays indicate that CRISPRa technologies can delineate the role of individual tumor suppressors. This would enable the development of tailored multiplexing strategies to reprogram multiple facets of the HCC phenotype. Among the TSGs tested, we found that CRISPRa-mediated upregulation of *HHIP* decreased HCC proliferation, confirming its role in promoting stemness [43–49] and reinforcing its role as a potential therapeutic target. Moreover, the TSG *TTC36* which is under-expressed in late-stage HCC [62–64], when upregulated by CRISPRa reduced cell migration, demonstrating a potential functional role in controlling cancer cell motility. We also show that CRISPRa-multiplexing of four genes (*HHIP*, *MT1M*, *PZP*, and *TTC36*) could potentially be harnessed to reduce both cell proliferation and migratory potential. Since Hep3B and HuH-7 cells are poorly tumorigenic in mouse models, future studies will require the testing of CRISPRa platforms in additional pre-clinical models, including patient-derived organoids (PDOs) [77] and patient-derived xenografts (PDXs) [78].

Finally, an ongoing challenge for the implementation of epigenetic manipulation in oncology is the targeted delivery of these CRISPRa platforms. One possible strategy is the use of liver-specific AAV8 [79], or the hit-and-run approaches for the delivery of the CRISPRa-encoded biomolecular components, such as in plasmid DNA [21], mRNA [80], or ribonucleoprotein complexes (RNPs) encapsulated in targeted non-toxic cell-released biological nanoparticles [81]. In this context, we recently described synthetic polymeric targeted formulations which can deliver plasmid DNA specifically into human HCC cells [82, 83].

## Conclusions

In summary, we have shown that CRISPRa technologies enable the selective activation “at will” of single as well as multiple TSGs silenced in HCC patients. This approach is associated with a significant normalization of the cancer cell phenotype. We have also developed an innovative toolbox that can drive specific and selective epigenetic manipulation of HCC that has the potential to be implemented as a precision oncology approach to treat this highly aggressive cancer.

## Methods

### Computational analyses

The computational scripts used in this study are available from the associated GitHub repository: https://github.com/jcursons/2022_Sgro_LIHC_tumour_suppressors.

### DNA methylation analysis

Illumina Infinium 850K methylation microarray data were imported into R and normalized using the Illumina Human MethylationEPIC Array manifest and missMethyl [84] R/Bioconductor packages. Data were SWAN-normalized [85], and probes were filtered on detection of *P* values before the calculation of *M* and *β* values. Differential expression analyses were performed on *β* values using edgeR [86].

### Clinical cancer data

Processed RNA-seq, miRNA abundance, and DNAme (probe-level *β*-value) data from the liver cancer and hepatocellular carcinoma (LIHC) cohort were a subset from The Cancer Genome Atlas (TCGA) Pan-Cancer Clinical Data Resource (TCGA-CDR) [87].

### The Cancer Genome Atlas (TCGA) data

Analysis of the TCGA liver cancer data was performed using Python (v 3.7.4) with the SciPy [88], pandas [89], Matplotlib [90], and NumPy [91] libraries. Processed TCGA molecular data were downloaded from https://gdc.cancer.gov/about-data/publications/pancanatlas and annotated with patient data from the TCGA-CDR [87] and the original TCGA-LIHC manuscript [12].

### Integrated-data cluster (iClust) inference

The original TCGA-LIHC manuscript [12] used an integrated data set with DNA copy number, gene methylation, miRNA abundance, and RNA abundance and performed clustering to identify 3 ‘iClusters’. A number of samples were excluded from this classification as they did not have data available across all of the associated platforms. To infer the iCluster values of unspecified samples for further analysis in this study, a differential gene expression analysis was performed between samples of known iClust values using limma and edgeR. Differentially expressed gene lists were used as signatures for gene set scoring with the *singscore* package [92], and scores for the three iClust gene sets were used for a *k*-nearest neighbors classification (*n*_neighbors_ = 30) with the scikit-learn [93] package. Samples were assigned to the iClust with the highest probability predicted by this classifier (Additional file 1: Fig. S1).

### RNA-seq and methylation data from the Cancer Cell Line Encyclopedia (CCLE)

We downloaded the RNA-seq count data (Feb 2020) and methylation data (Aug 2020) of liver cancer cell lines from the Cancer Cell Line Encyclopedia (CCLE) (https://data.broadinstitute.org/ccle/) and used R version 4.0.5 to compare the two data types. For the RNA-seq data, we retained genes with a count > 5 in at least five cell lines, performed TMM normalization [94], calculated log RPKM values using the rpkm function in the edgeR package (v 3.32.1) [86], and used the gene length information from GENCODE version 19. The tidyr (v 1.1.3, including ggplot2) package was used for data manipulation and visualization.

### Cell culture

Hep3B and HuH-7 HCC cell lines (a gift from Professor Peter J. Leedman’s Laboratory) were cultured in DMEM low glucose-pyruvate supplemented containing 10% HyClone fetal bovine serum (FBS) (GE Healthcare Life Sciences, Cat. No. SH30084.03) and 1% antibiotic–antimycotic (Gibco, Cat. No. 15240062). HEK293T cells (ATCC, Cat. No. CRL-3216) were grown in DMEM high glucose-pyruvate containing 10% FBS and 1% antibiotic–antimycotic. The immortalized human liver cell line THLE-3 (ATCC, Cat. No. CRL-1123) was grown in BEBM supplemented with BEGM Bullet Kit (Lonza, Cat. No. CC-3170), from which we omitted the gentamycin/amphotericin and epinephrine, and to which we added 5 ng/mL human epidermal growth factor (EGF) recombinant protein (Gibco, Cat. No. PHG0311), 70 ng/mL *O*-Phosphorylethanolamine (Sigma-Aldrich, Cat. No. P0503), and 10% FBS. These cells were maintained in flasks pre-coated with a mixture of 0.01 mg/mL human fibronectin (Gibco, Cat. No. 33016015), 0.03 mg/mL bovine collagen type I (Australian Biosearch, Cat. No. 5005-B), and 0.01 mg/mL bovine serum albumin (Sigma-Aldrich, Cat. No. A9647) dissolved in BEBM medium (Lonza). All cell lines used in this study were *Mycoplasma*-free and maintained at 37 °C and 5% CO_2_.

### Normal hepatocytes

Total RNA from a donor’s healthy normal liver was extracted using the RNeasy Plus Mini Kit (Qiagen, Cat. No. 74134) as per the manufacturer’s instructions, under the conditions of the ethical approval (RGS0000001834) granted to Professor George C.T. Yeoh by the Human Research Ethics Committee at the Sir Charles Gairdner Hospital, Perth, Western Australia.

### Drug treatments

Hep3B and HuH-7 cells were seeded in complete medium in 6-well plates at a concentration of 6 × 10^4^ for treatments with 5-aza-2′-deoxycytidine, i.e., decitabine (Selleckchem, Cat. No. S1200, Batch No.09) or 15 × 10^4^ for treatments with suberoylanilide hydroxamic acid (SAHA), i.e., vorinostat (Selleckchem, Cat. No. S1047, Batch No.11). Twenty-four hours later, the cells were treated with 5-aza-2′-deoxycytidine or SAHA at concentrations of 1, 2.5, 5, 7.5, and 10 μM or 1, 2, and 4 μM for 72 or 48 h, respectively. The culture medium was replaced daily, and dimethyl sulfoxide (DMSO) was used as vehicle control. Cells were subsequently collected for RNA extraction.

### gRNA designing and off-target identification

Four to five candidate gRNA sequences for gene activation, with optimized on- and off-target scores [33, 34] (https://benchling.com/), were selected within the proximal promoter and CpG islands of each targeted TSG (Additional file 10: Table S1). To search for potential genomic off-target binding sites with three mismatches or less to the cognate most active TSG-targeting gRNA sequences, the algorithm Cas-OFFinder [35] was implemented. The genomic location for each off-target sequence was bioinformatically mapped on the UCSC Genome Browser with the integrated information relevant to the regulation of transcription from the ENCODE project. The off-target sequences found in proximity to gene regulatory elements were assessed by qRT-PCR in transiently transfected and lentivirally transduced cells (Additional file 4, 5: Fig. S4 and Additional file 16: Data S5).

### Plasmids

The plasmids utilized for transient transfections in this study were pcDNA™3.1 (+) empty vector Zeo backbone (Invitrogen, Cat. No. V790-20); pUC19 sgRNA cloning backbone with MS2 loops at tetraloop and stem-loop 2 that contains BbsI sites for insertion of spacer sequences (Addgene plasmid # 61424, a gift from Feng Zhang); MS2-p65-HSF1_GFP (Addgene plasmid # 61423, a gift from Feng Zhang); SP-dCas9-VPR (Addgene plasmid # 63798, a gift from George Church); pdCas9-Tet1-CD and pcDNA3.1-MS2-Tet1-CD (Addgene plasmids # 83340 and # 83341, respectively, a gift from Ronggui Hu); and pcDNA-dCas9 (Addgene plasmid # 47106, a gift from Charles Gersbach).

The plasmids utilized for lentiviral transductions in this study were pMD2.G (VSV-G envelope expressing plasmid) and the third generation packaging pMDLg/pRRE (GAG and POL expressing plasmid) (Addgene plasmids # 12259 and # 12251, respectively, a gift from Didier Trono), pLV hU6-sgRNA hUbC-dCas9-VPR-T2A-Puro [19], and pLV hU6-sgRNA hUbC-dCas9-TET1-CD-T2A-Puro (this paper, see Molecular cloning section).

### Molecular cloning

The pLV hU6-sgRNA hUbC-dCas9-TET1-CD-T2A-Puro lentiviral vector was generated from the pLV hU6-sgRNA hUbC-dCas9-KRAB-T2A-Puro (Addgene plasmid # 71236, a gift from Charles Gersbach) by replacing the KRAB domain with the catalytic domain of TET-1 from Fuw-dCas9-Tet1CD (Addgene plasmid # 84475, a gift from Rudolf Jaenisch).

For each gRNA, sense and anti-sense customized DNA oligonucleotides (IDT, Integrated DNA Technologies) were annealed and ligated, using T4 DNA ligase (Promega, Cat. No. M1801), into the BbsI recognition site of the transient pUC19 sgRNA (MS2) cloning backbone or into the BsmBI recognition site of the lentiviral vectors pLV hU6-sgRNA hUbC-dCas9-VPR-T2A-Puro and pLV hU6-sgRNA hUbC-dCas9-TET1-CD-T2A-Puro.

### Transient transfection

Hep3B and HuH-7 cells were seeded in a complete culture medium at a density of 3.5 × 10^5^ cells/well in 6-well plates 18 h prior to transfection using Lipofectamine 3000 (Invitrogen, Cat. No. L3000001). The next day, the cells were incubated with a transfection mix containing transfection reagents and 2.55 μg of plasmid DNA in Opti-MEM (Gibco), according to the manufacturer’s protocol. The culture medium containing the transfection mix was removed after 4 h and exchanged for fresh culture medium. Cells were collected 48 h post-transfection to assess mRNA upregulation of VPR/gRNA-MS2-MCP-p65-HSF1 activator constructs based on our previous work [18]. Cells were also harvested 72 h post-transfection to determine mRNA modulation when tiling the proximal promoter with TET1-CD systems, and 96 h post-transfection for RNA extraction and DNA methylation assays to provide additional cell division cycles for DNA demethylation activities when employing TET1-CD-based constructs, as previously described [67].

### Quantitative real-time PCR (qRT-PCR)

Total RNA was extracted from cultured, transfected, and transduced cells using QIAzol Lysis Reagent (Qiagen) as per the manufacturer’s protocol. 1000 ng of extracted RNA was used for cDNA synthesis using a High-Capacity cDNA Reverse Transcription Kit or a TaqMan™ MicroRNA Reverse Transcription Kit (Applied Biosystems). Relative quantification of the transcript expression was obtained by qRT-PCR using TaqMan probes in a ViiA™ 7 Real-Time PCR System (Applied Biosystems). The TaqMan probes utilized are listed in Additional file 11: Table S2. The data were analyzed using the ΔΔ*C*_*t*_ method [95] with *GAPDH*, *GUSB*, *PPIA*, and *RNU6B* as housekeeping genes for normalization.

### Genomic DNA purification

Genomic DNA was obtained from Hep3B cells seeded at a density of 3.5 × 10^5^ cells/well in 6-well plates and transiently transfected for 96 h. Cells were washed in Dulbecco’s PBS (DPBS), collected, and centrifuged at 1000 rpm for 5 min at room temperature. Pelleted cells were washed in DPBS and centrifuged again. Cell pellets were then processed using a Monarch^®^ Genomic DNA Purification Kit (NEB, Cat. No. T3010L) according to the manufacturer’s protocol. Genomic DNA was shipped at room temperature to the AGRF (Australian Genome Research Facility, Melbourne) for DNA methylation analysis using an Illumina Infinium 850K methylation microarray. Bisulfite-converted DNA was amplified, fragmented, and hybridized to Illumina Infinium MethylationEPIC BeadChip Kit using standard Illumina protocol.

### Lentiviral production and transduction

Hep3B cells were transduced with lentivirus to constitutively co-expressed *Sp*dCas9-VPR [19] or *Sp*dCas9-TET1-CD (this paper) along with the tumor suppressor gene (TSG)-targeting gRNAs (CRISPRa). Lentiviral particles were generated by transfecting HEK293T cells with 4.5 μg of CRISPRa lentiviral expression plasmid, 1.54 μg of pMD2.G (VSV-G envelope expressing plasmid), and 2.88 μg of pMDLg/pRRE (GAG and POL expressing plasmid) mixed with 50 μL of Lipofectamine 2000 (Thermo Fisher Scientific, Cat. No 11668019) in Opti-MEM (Gibco). After 4 h, the transfection medium was replaced with HEK293T medium. The supernatants containing lentivirus were collected for transduction 42, 50, and 66 h after the first medium exchange, and cleared from residual producer cells by filtration through 0.22 μm hydrophilic PVDF membrane syringe filters (Millipore). The cationic polymer polybrene (Sigma-Aldrich, Cat. No. 107689) was added to the filtered viral supernatants at a concentration of 8 μg/mL to promote transduction. Hep3B cells, plated in 10 cm plates, were transduced three times with the lentiviral particles, for a period of 8–12 h each time. For the combination of gRNAs (MIX 4G) or for genome multiplexing (MIX 4 genes), equal volumes of lentiviral particles were added to the host cells. Eight hours after the last transduction, the medium was replaced with a fresh medium to remove the virus. Transduced cells with lentivirus containing a puromycin-resistance gene were treated with 1.75 μg/mL puromycin (Gibco, Cat. No. A1113803) 72 h after transduction to initiate selection.

### Immunofluorescence

Hep3B cells lentivirally transduced with *Sp*dCas9-VPR (CRISPRa) were seeded at a density of 2.5 × 10^4^ cells onto 13-mm glass coverslips pre-treated with poly-L-lysine hydrobromide (Sigma-Aldrich, Cat. No. P6282). Twenty-four hours after seeding, the adhered cells were fixed with 4% Pierce™ Formaldehyde Methanol-free (Thermo Fisher Scientific, Cat. No. 28908) in DPBS for 20 min at room temperature and washed twice with DPBS. Fixed cells were blocked with 5% normal goat serum (Invitrogen, Cat. No. 31872) and 0.3% Triton X-100 (Sigma-Aldrich) in DPBS for 1 h at room temperature, then incubated with α-Ki-67 mouse monoclonal antibody (Cell Signaling Technology, Cat. No. 9449, 1:500) in diluent buffer (1% BSA and 0.3% Triton X-100 in DPBS) overnight at 4 °C. The next day, the cells were washed and incubated with a goat α-mouse secondary Alexa Fluor 488-conjugated antibody (Thermo Fisher Scientific, Cat. No. A11001, 1:500) for 2 h at room temperature. The coverslips were mounted on slides with SlowFade™ Diamond Antifade Mountant (Thermo Fisher Scientific, Cat, No. S36963) and the percentage of cells positive for Ki-67 versus the total number of cells counterstained with Hoechst 33258 (Sigma-Aldrich, Cat. No. 94403, 1:5,000) was assessed in 12 fields of view from images acquired with an Olympus DP71 fluorescence microscope.

For protein detection of the four genes targeted by *Sp*dCas9-VPR (CRISPRa), the primary antibodies used were α-HHIP rabbit polyclonal (Invitrogen, Cat. No. PA5-22242, 1:400), α-MT1M rabbit polyclonal (ProteinTech, Cat. No. 17281-1-AP, 1:200), α-PZP rabbit polyclonal (Abcam, Cat. No. ab233166, 1:20), α-TTC36 rabbit polyclonal (Abcam, Cat. No. ab122507, 1:300), and α-*Sp*Cas9 mouse monoclonal [7A9-3A3] (Abcam, Cat. No. ab191468, 1:500). The secondary antibodies used were Alexa Fluor 488-conjugated goat α-mouse (Thermo Fisher Scientific, Cat. No. A11001, 1:500) and Alexa Fluor 594-conjugated goat α-rabbit (Thermo Fisher Scientific, Cat. No. A11012, 1:500); Hoechst 33258 was used to stain the cell nuclei. Images were acquired by a confocal fluorescence Nikon Ti-E inverted microscope using a Nikon Plan Apo VC 60x/1.40 Oil OFN25 DIC N2 objective and collected using NIS-C Elements Software.

### Cell viability assay

Hep3B cells lentivirally transduced with CRISPRa were seeded at a density of 5 × 10^3^ cells/well in complete medium in 96-well white-bottom plates (Greiner) and processed after 4, 24, 48, and 72 h with CellTiter-Glo^®^ 2.0 (Promega, Cat. No. G9241) luminescence assay protocol to determine cell viability. The luminescence was measured using a CLARIOstar plate reader (BMG Labtech, Mornington, VIC, Australia). The luciferase measurements were normalized to CRISPRa NO G control.

### Migration assay

Hep3B cells lentivirally transduced with CRISPRa were seeded at the concentration of 5 × 10^4^ in serum-free medium in the inner chamber of Costar^®^ Transwell cell culture inserts (6.5 mm diameter, 8 μm pore size, Corning, Cat. No. 3422). A complete medium containing 10% FBS was added to the outer chamber as a chemoattractant, and the cells were incubated for 48 h. The inserts were stained as per protocol using a staining solution containing 0.5% crystal violet dissolved in 25% methanol and sterile water for 10 min. Images from 12 fields of view were acquired with an inverted Leica light microscope to quantify the percentage of cells that had migrated, based on biological triplicates, using ImageJ software.

### Statistical analyses

All data were derived from multiple experiments conducted at least in triplicate. Statistical analyses were performed with Prism 9 (GraphPad Software, La Jolla, CA, USA) and are detailed in the figure legends. For iCluster signature derivation, differential gene expression was performed in R (v 3.6.1; https://www.r-project.org/) using limma [96] with a Benjamini–Hochberg correction and TREAT criteria (logFC > 1.5, adj. *P* value < 0.05) [97]. The identification of differentially methylated probes was performed using limma with a Benjamini–Hochberg correction.

## Supplementary Information

**Additional file 1: Figure S1. Related to Fig.** 1**. A** Scatter plots showing the associations between different integrated-data cluster (iClust) scores of individual tumor samples. Tumor samples with an iClust value specified in the original TCGA manuscript are shown (*dark blue*, *dark orange*, and *dark green*), together with inferred iClust annotations (*light blue*, *light orange*, and *light green*) obtained through a k-nearest neighbors’ classification (further details given in “Methods”).

**Additional file 2: Figure S2. Related to Fig.** 1**. A** The percentage of patients who have evidence of gene hypermethylation and no evidence of gene loss/deletion within each iCluster group (iClust1: *blue*; iClust2: *orange*; and iClust3: *green*) and across all patients (*black*). **B** The percentage of patients who have evidence of gene hypermethylation and no evidence of gene loss/deletion in at least one gene of the listed set, within each iCluster group and across all patients. Genes were defined as hypermethylated if their corresponding probe β-value was greater than all matched-adjacent/normal liver tissue samples

**Additional file 3: Figure S3. Related to Fig.** 2**. A** Anticorrelation between RNA abundance (from RNA-seq data) and DNA methylation data of the available genes (*CPS1*, *HHIP*, *MT1E*, *MT1M*, *PSAT1*, *PTGR1*, *TMEM106A*, and *TTC36*) for Hep3B and HuH-7 HCC cell lines from the Cancer Cell Line Encyclopedia. RPKM, Reads Per Kilobase Million.

**Additional file 4: Figure S4 (part 1). Related to Figs.** 3–7**. A** Off-target analysis of the ten TSGs reactivated in this study, using the most potent gRNAs. The potential off-target genes (*MMP11*, *MOCOS*, *PIR*, *SEMA5A*, *TMEM14C*, *HRH1*, *NACC2*, *NONO*, *PPA1*, *RBM39*, *NKAIN3*, and *ABCC3*) were found in proximity to genomic regulatory regions.

**Additional file 5: Figure S4 (part 2). Related to Figs.** 3–7**. CRISPRa is highly specific in hit-and-run and lentiviral approaches in HCC cell lines. A-K** The potential off-target genes (*MMP11*, *MOCOS*, *PIR*, *SEMA5A*, *TMEM14C*, *HRH1*, *NACC2*, *NONO*, *PPA1*, *RBM39*, *NKAIN3*, and *ABCC3*) found in proximity to genomic regulatory regions for the most potent gRNAs utilized were assessed by qRT-PCR 48 h post-transfection, employing *Sp*dCas9-VPR and MS2-MCP-p65-HSF1 system in HuH-7 (**A**), (**B**), (**C**), (**E**) and Hep3B cells (**F**), (**H**), (**J**); and in stable Hep3B cell lines expressing *Sp*dCas9-VPR (**D**), (**G**), (**I**), (**K**). Relative gene expression, expressed as fold change, was normalized to cells transfected with empty vector control (EV) or to cells transduced with *Sp*dCas9-VPR NO G, and compared to either EV or NO G conditions for statistical analysis. Data presented as means ± SEM (*n* = 3), and *P*-values were determined by two-way ANOVA with Dunnett's multiple comparisons test for panels (**A**), (**B**), (**C**), (**E**), (**F**), (**H**), and (**J**) (**A**: ***P = 0.0001; **B**, **C**, **E**, **F**, and **H**: ****P < 0.0001; **J**: ***P = 0.0003, ****P < 0.0001); or by two-way ANOVA with Šídák's multiple comparisons test for panels **D**, **G**, **I**, and **K** (****P < 0.0001).

**Additional file 6: Figure S5. Related to Fig.** 5**. Tiling the promoter of MT1M and TTC36 tumor suppressor genes with a CRISPRa toolbox in Hep3B HCC cells. A-B** Fold change in *MT1M* (**A**) and *TTC36* (**B**) mRNA expression evaluated by qRT-PCR 72 h after transient transfection in Hep3B cells. Cells were transfected with combinations of CRISPRa along with a mix of four gRNAs (MIX 4G), or with no gRNA (NO G) as control. Relative gene expression was normalized and compared to cells transfected with empty vector control (EV) for statistical analysis. Data presented as means ± SEM (*n* = 3), and *P*-values were determined by one-ANOVA with Dunnett's multiple comparisons test (****P < 0.0001). *Sp*dCas9 *Streptococcus pyogenes* deactivated Cas9 protein adopted for epigenome engineering, VPR VP64, p65, Rta, MS2 RNA aptamer, MCP MS2-coat protein, HSF1 heat shock factor 1, TET1-CD Ten-Eleven Translocation methylcytosine dioxygenase 1-catalytic domain, MIX 4G combination of four gRNAs.

**Additional file 7: Figure S6. Related to Fig.** 5**. Transcriptional reactivation of HHIP gene by novel CRISPRa combinations does not correlate with changes in promoter DNA methylation in transiently transfected Hep3B cells. A** Fold change in *HHIP* mRNA expression evaluated by qRT-PCR 96 h after transient transfection in Hep3B cells. Cells were transfected with *Sp*dCas9-VPR and MS2-MCP-p65-HSF1, or with *Sp*dCas9-TET1-CD and MS2-MCP-p65-HSF1, along with gRNA G4 or with no gRNA (NO G) as control. Relative gene expression was normalized and compared to cells transfected with empty vector control (EV) for statistical analysis. Data presented as means ± SEM (*n* = 3), and *P*-values were determined by unpaired *t-*test (***P = 0.0001, ****P < 0.0001). **B** Heatmap showing *HHIP* promoter DNA methylation in Hep3B cells transiently transfected with the novel CRISPRa combinations. Data from Illumina Infinium 850 K methylation EPIC microarrays. For each probe (listed at left), the difference in β-value from the average is shown (at left; *green-purple* color map) together with the average probe β-value (at center; *blue-yellow* color map), and the adjusted *P*-value for differential methylation between gRNA G4 and NO G for the two combinations tested (at right; *white-purple* color map).

**Additional file 8: Figure S7. Related to Fig.** 5**. Lentiviral transduction of SpdCas9-TET1-CD or SpdCas9-VPR targeting HHIP or MT1M genes in Hep3B cells. A** Schematic representation of the “all-in-one” lentiviral vectors, *Sp*dCas9-TET1-CD and *Sp*dCas9-VPR, for the constitutive co-expression of a gRNA and TET1-CD or VPR, C-terminally fused to *Sp*dCas9. **B** Fold change in *HHIP* mRNA expression assessed by qRT-PCR at 18, 30, and 40 days in Hep3B cells constitutively co-expressing *Sp*dCas9-TET1-CD and the combination of four gRNAs (MIX 4G) targeting *HHIP* promoter. **C** Fold change in *MT1M* mRNA expression assessed by qRT-PCR at 20 days in Hep3B cells lentivirally co-transduced with *Sp*dCas9-TET1-CD and individual gRNAs (G1, G2, G3, and G4) or the MIX 4G; or with *Sp*dCas9-VPR and the most potent gRNA (G1). Data presented as means ± SEM (*n* = 3), compared to *Sp*dCas9-TET1-CD NO G, and *P*-values were determined by two-way ANOVA with Šídák's multiple comparisons test for panel (**B**), and by two-way ANOVA with Dunnett's multiple comparisons test for panel (**C**) (****P < 0.0001). *Sp*dCas9 *Streptococcus pyogenes* deactivated Cas9 protein adopted for epigenome engineering, TET1-CD Ten-Eleven Translocation methylcytosine dioxygenase 1-catalytic domain, VPR VP64, p65, Rta, hU6 RNA polymerase III promoter for human U6 snRNA, hUbC human ubiquitin C promoter, 3xFLAG three tandem FLAG^®^ epitope tags, followed by an enterokinase cleavage site, SV40NLS nuclear localization signal of SV40 (simian virus 40) large T antigen, T2A 2A peptide from *Thosea asigna* virus capsid protein, cleavable linker, PuroR puromycin *N*-acetyltransferase gene that confers resistance to puromycin, MIX 4G combination of four gRNAs, ns not significant.

**Additional file 9: Figure S8. Related to Fig.** 7**. Phenotypic reprogramming in Hep3B HCC cells lentivirally transduced with SpdCas9-VPR targeting and upregulating MT1M with G1, PZP with G2, TTC36 with G3, or with NO G as control. A-C** Cell viability determined using a luminescence assay (CellTiter-Glo^®^). Data shown as fold change compared to *Sp*dCas9-VPR NO G at 24, 48, and 72 h, presented as means ± SEM (*n* = 3), and *P*-values were determined by unpaired *t-*test with Welch's correction (From left to right: (**A**), ***P = 0.0001, **P = 0.0056; (**B**), **P = 0.0024, **P = 0.0011; and (**C**), ****P < 0.0001, *P = 0.0310). **D** Cell migration assessed by the Boyden chamber assay. Data normalized to *Sp*dCas9-VPR NO G, presented as means ± SEM (*n* = 3), and *P*-values were determined by unpaired *t-*test. ns not significant.

**Additional file 10: Table S1**. **Related to Figs.** 3–7** and Figures S4-S8**. gRNAs designed for gene reactivation by CRISPRa technology. Details include gRNA and PAM sequences, the DNA strand targeted, the distance from the TSS, and the genomic annotations.

**Additional file 11: Table S2**. **Related to Figs.** 2–7** and Figures S4-S7**. TaqMan gene expression assays utilized for qRT-PCR experiments.

**Additional file 12: Data S1**. **Related to Fig.** 1. Assigned 'integrated cluster' (iClust) groups for TCGA liver cancer samples where available (from the original TCGA-LIHC manuscript), together with inferred iClusters for unspecified samples, as determined by clustering of gene set scores (as shown in Figure S1; further details given in “Methods”).

**Additional file 13: Data S2**. **Related to Fig.** 1. mRNA transcript abundance, probe methylation (beta) values, and copy number variation data for all TCGA liver samples (including matched adjacent normal liver tissue). Corresponds to pre-transformed data shown in Fig. 1.

**Additional file 14: Data S3**. **Related to Fig.** 1. z-score normalized mRNA transcript abundance, probe methylation (beta) values, and copy number variation for all TCGA liver samples (including matched adjacent normal liver tissue). Corresponds to data shown in Fig. 1.

**Additional file 15: Data S4**. **Related to Fig.** 2**, Fig.** 3**, Fig.** 4**, and Fig.** 6. Comparison of the fold change in mRNA expression for the tumor suppressor genes targeted individually or multiplexed by CRISPRa system (*Sp*dCas9-VPR and MS2-MCP-p65-HSF1) versus treatments with the epi-drugs (5-aza and SAHA) in Hep3B and HuH-7 cells.

**Additional file 16: Data S5**. **Related to Figs.** 3, 4, and 7. Comprehensive bioinformatic analysis of predicted potential off-targets for the ten TSGs targeted by CRISPRa technology utilizing the most active gRNAs.

**Additional file 17: Data S6**. **Related to Figure S6**. Probe methylation (beta) values for *HHIP* in transiently transfected Hep3B cells with *Sp*dCas9-VPR and MS2-MCP-p65-HSF1, or *Sp*dCas9-TET1-CD and MS2-MCP-p65-HSF1 targeting *HHIP* promoter.

## Data Availability

All data generated or analyzed during this study are included in this article. DNAme data generated in this study are available from the Gene Expression Omnibus page (https://www.ncbi.nlm.nih.gov/geo/) under accession number GSE211837. Patient data were downloaded from The Cancer Genome Atlas (https://gdc.cancer.gov/about-data/publications/pancanatlas) and Cancer Cell Line Encyclopedia (CCLE) data were downloaded from the Cancer Dependency Map (https://depmap.org/portal/download/).
