# Supplementary figures and images for: Epigenetic reactivation of tumor suppressor genes with CRISPRa technologies as precision therapy for hepatocellular carcinoma
Source: Clin Epigenetics. 2023 Apr 29;15:73. doi: 10.1186/s13148-023-01482-0 (PMC10149030; doi:10.1186/s13148-023-01482-0)

Supplementary Figure S1

A

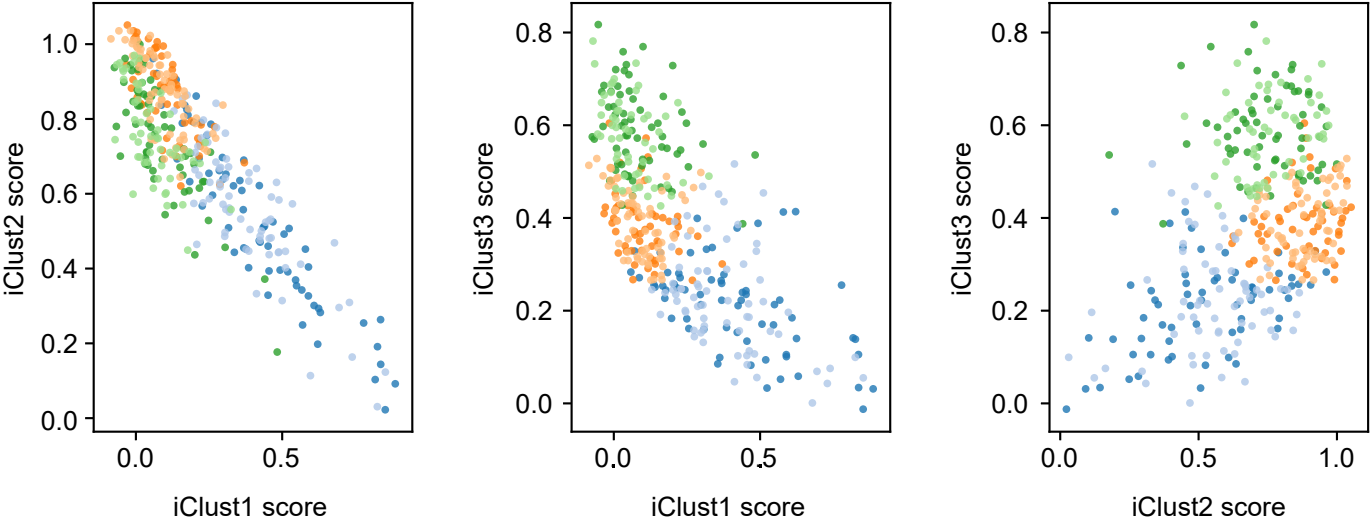

Supplement: Supplementary file 1 — Additional file 1: Figure S1. Related to Fig. 1. A Scatter plots showing the associations between different integrated-data cluster (iClust) scores of individual tumor samples. Tumor samples with an iClust value specified in the original TCGA manuscript are shown (dark blue, dark orange, and dark green), together with inferred iClust annotations (light blue, light orange, and light green) obtained through a k-nearest neighbors’ classification (further details given in “Methods”). [file 13148_2023_1482_MOESM1_ESM.pdf]

Supplementary Figure S2

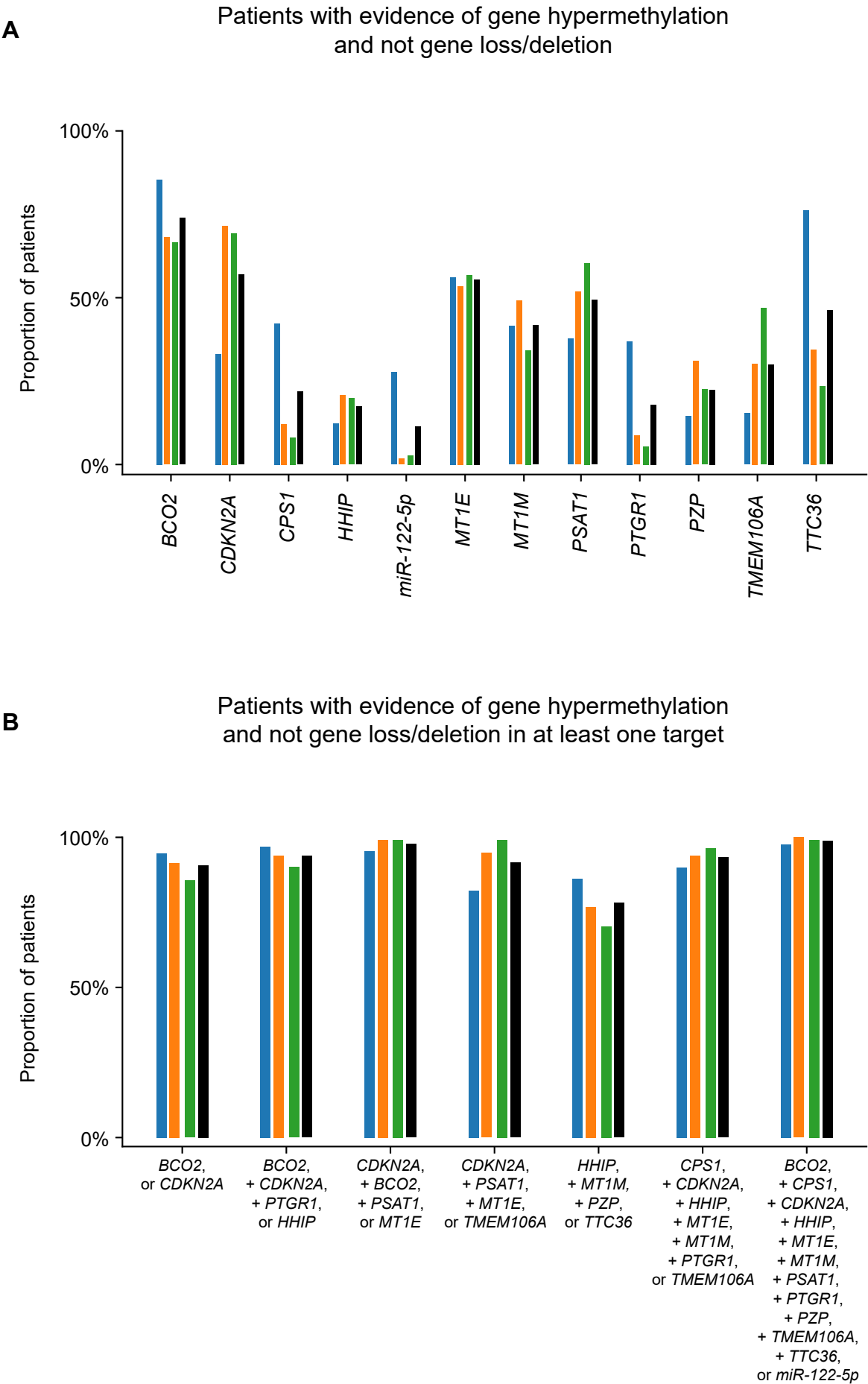

Supplement: Supplementary file 2 — Additional file 2: Figure S2. Related to Fig. 1. A The percentage of patients who have evidence of gene hypermethylation and no evidence of gene loss/deletion within each iCluster group (iClust1: blue; iClust2: orange; and iClust3: green) and across all patients (black). B The percentage of patients who have evidence of gene hypermethylation and no evidence of gene loss/deletion in at least one gene of the listed set, within each iCluster group and across all patients. Genes were defined as hypermethylated if their corresponding probe β-value was greater than all matched-adjacent/normal liver tissue samples [file 13148_2023_1482_MOESM2_ESM.pdf]

Supplementary Figure S3

A

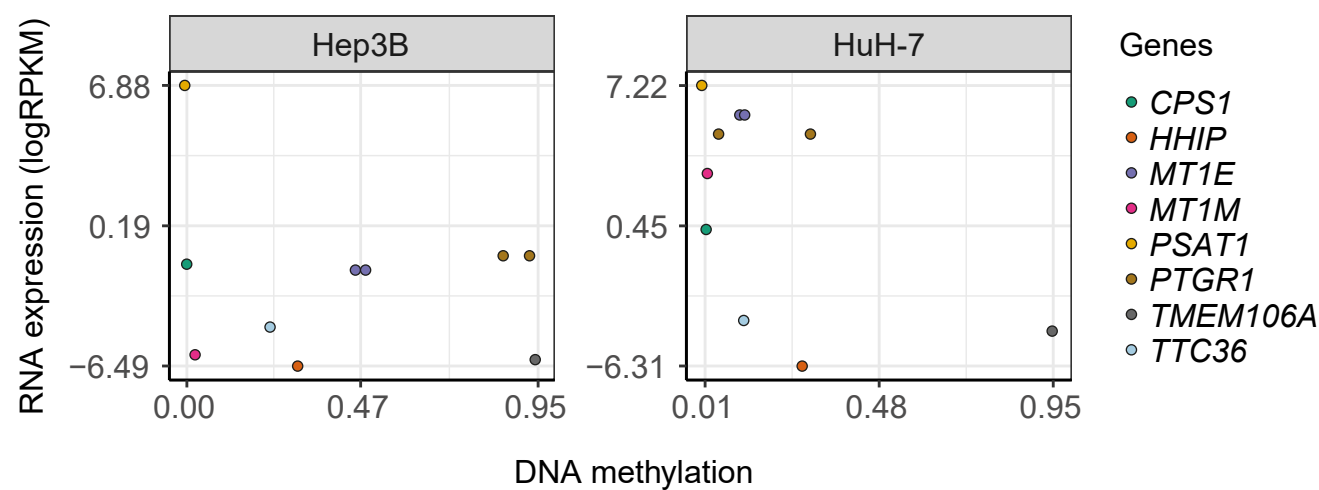

Supplement: Supplementary file 3 — Additional file 3: Figure S3. Related to Fig. 2. A Anticorrelation between RNA abundance (from RNA-seq data) and DNA methylation data of the available genes (CPS1, HHIP, MT1E, MT1M, PSAT1, PTGR1, TMEM106A, and TTC36) for Hep3B and HuH-7 HCC cell lines from the Cancer Cell Line Encyclopedia. RPKM, Reads Per Kilobase Million. [file 13148_2023_1482_MOESM3_ESM.pdf]

Supplementary Figure S4 (part 2)

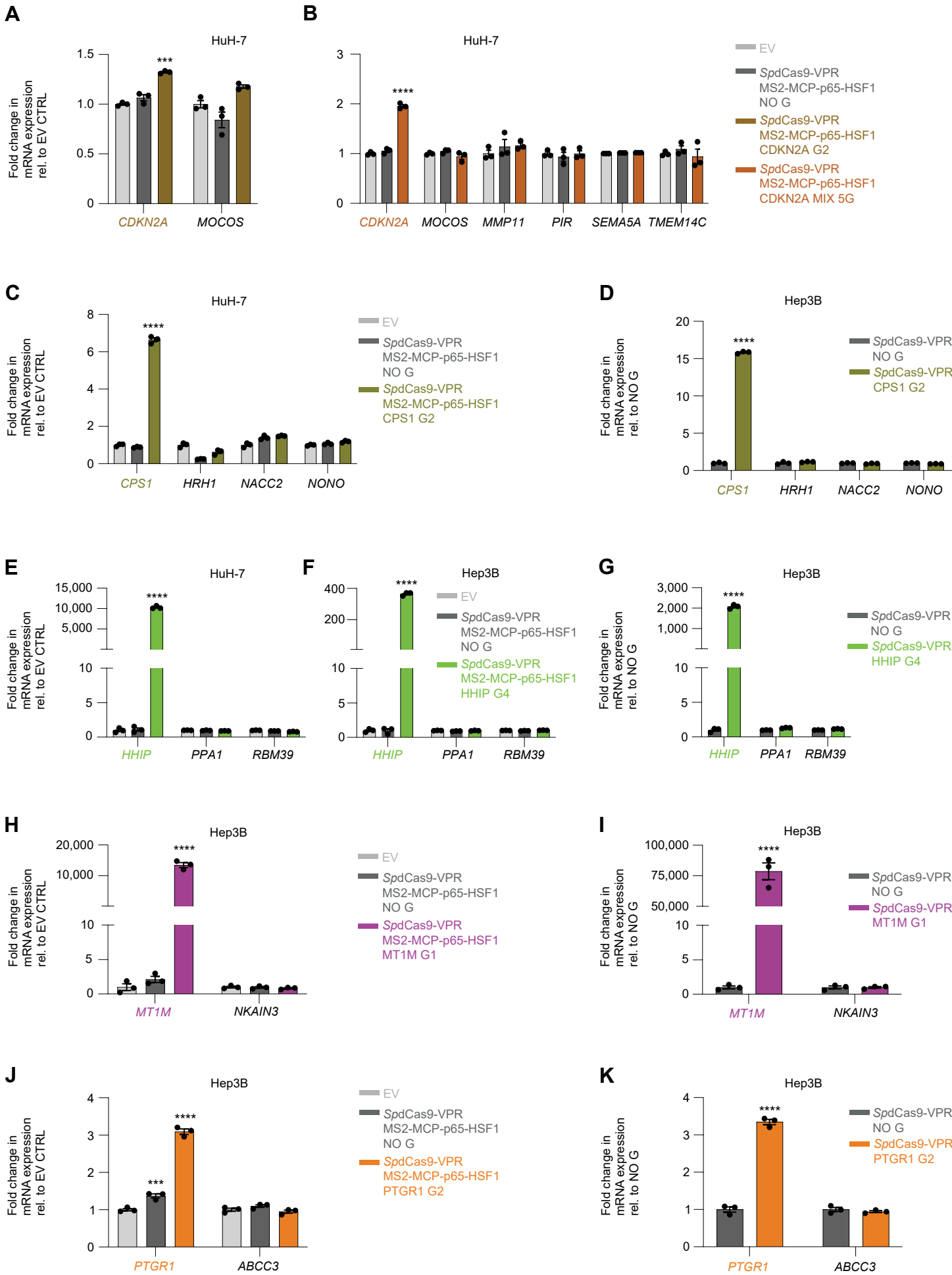

Supplement: Supplementary file 5 — Additional file 5: Figure S4 (part 2). Related to Figs. 3–7. CRISPRa is highly specific in hit-and-run and lentiviral approaches in HCC cell lines. A-K The potential off-target genes (MMP11, MOCOS, PIR, SEMA5A, TMEM14C, HRH1, NACC2, NONO, PPA1, RBM39, NKAIN3, and ABCC3) found in proximity to genomic regulatory regions for the most potent gRNAs utilized were assessed by qRT-PCR 48 h post-transfection, employing SpdCas9-VPR and MS2-MCP-p65-HSF1 system in HuH-7 (A), (B), (C), (E) and Hep3B cells (F), (H), (J); and in stable Hep3B cell lines expressing SpdCas9-VPR (D), (G), (I), (K). Relative gene expression, expressed as fold change, was normalized to cells transfected with empty vector control (EV) or to cells transduced with SpdCas9-VPR NO G, and compared to either EV or NO G conditions for statistical analysis. Data presented as means ± SEM (n = 3), and P-values were determined by two-way ANOVA with Dunnett's multiple comparisons test for panels (A), (B), (C), (E), (F), (H), and (J) (A: ***P = 0.0001; B, C, E, F, and H: ****P < 0.0001; J: ***P = 0.0003, ****P < 0.0001); or by two-way ANOVA with Šídák's multiple comparisons test for panels D, G, I, and K (****P < 0.0001). [file 13148_2023_1482_MOESM5_ESM.pdf]

Supplementary Figure S5

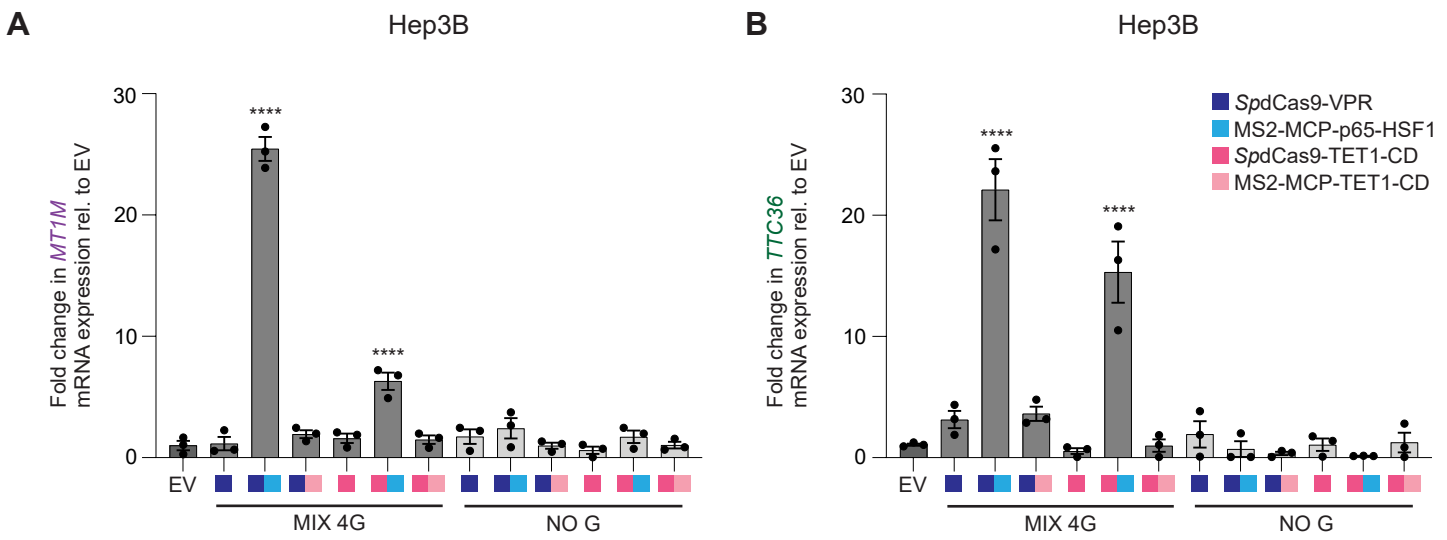

Supplement: Supplementary file 6 — Additional file 6: Figure S5. Related to Fig. 5. Tiling the promoter of MT1M and TTC36 tumor suppressor genes with a CRISPRa toolbox in Hep3B HCC cells. A-B Fold change in MT1M (A) and TTC36 (B) mRNA expression evaluated by qRT-PCR 72 h after transient transfection in Hep3B cells. Cells were transfected with combinations of CRISPRa along with a mix of four gRNAs (MIX 4G), or with no gRNA (NO G) as control. Relative gene expression was normalized and compared to cells transfected with empty vector control (EV) for statistical analysis. Data presented as means ± SEM (n = 3), and P-values were determined by one-ANOVA with Dunnett's multiple comparisons test (****P < 0.0001). SpdCas9 Streptococcus pyogenes deactivated Cas9 protein adopted for epigenome engineering, VPR VP64, p65, Rta, MS2 RNA aptamer, MCP MS2-coat protein, HSF1 heat shock factor 1, TET1-CD Ten-Eleven Translocation methylcytosine dioxygenase 1-catalytic domain, MIX 4G combination of four gRNAs. [file 13148_2023_1482_MOESM6_ESM.pdf]

Supplementary Figure S6

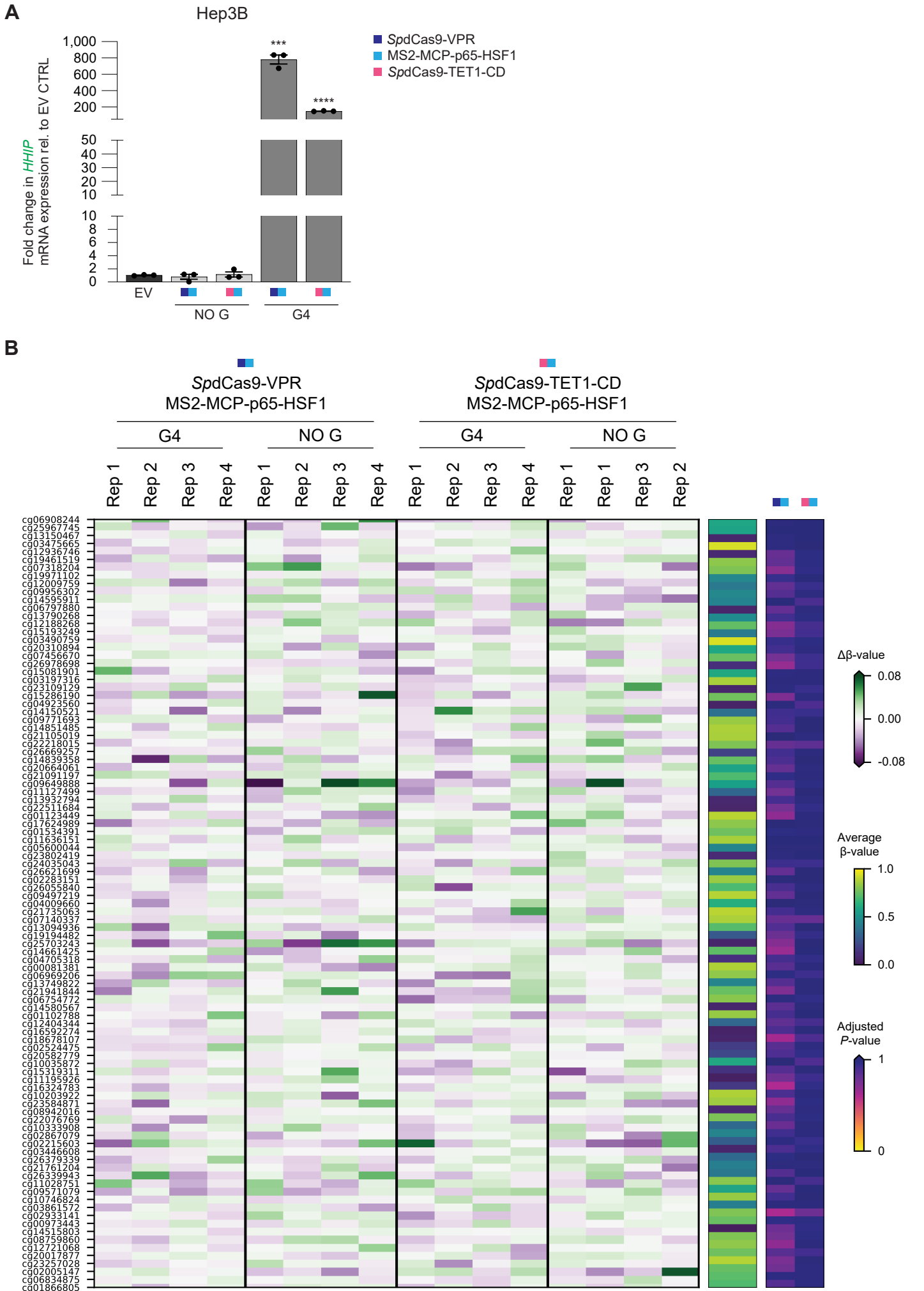

Supplement: Supplementary file 7 — Additional file 7: Figure S6. Related to Fig. 5. Transcriptional reactivation of HHIP gene by novel CRISPRa combinations does not correlate with changes in promoter DNA methylation in transiently transfected Hep3B cells. A Fold change in HHIP mRNA expression evaluated by qRT-PCR 96 h after transient transfection in Hep3B cells. Cells were transfected with SpdCas9-VPR and MS2-MCP-p65-HSF1, or with SpdCas9-TET1-CD and MS2-MCP-p65-HSF1, along with gRNA G4 or with no gRNA (NO G) as control. Relative gene expression was normalized and compared to cells transfected with empty vector control (EV) for statistical analysis. Data presented as means ± SEM (n = 3), and P-values were determined by unpaired t-test (***P = 0.0001, ****P < 0.0001). B Heatmap showing HHIP promoter DNA methylation in Hep3B cells transiently transfected with the novel CRISPRa combinations. Data from Illumina Infinium 850 K methylation EPIC microarrays. For each probe (listed at left), the difference in β-value from the average is shown (at left; green-purple color map) together with the average probe β-value (at center; blue-yellow color map), and the adjusted P-value for differential methylation between gRNA G4 and NO G for the two combinations tested (at right; white-purple color map). [file 13148_2023_1482_MOESM7_ESM.pdf]

Supplementary Figure S7

A

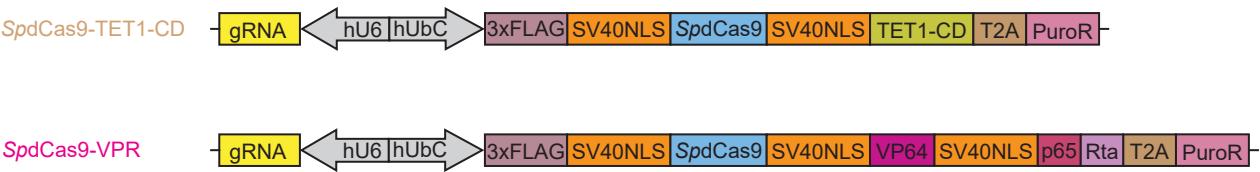

B

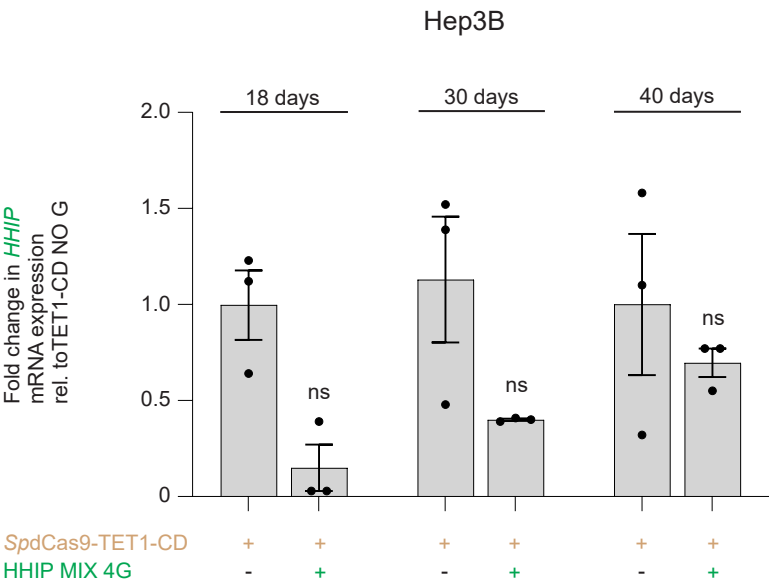

C

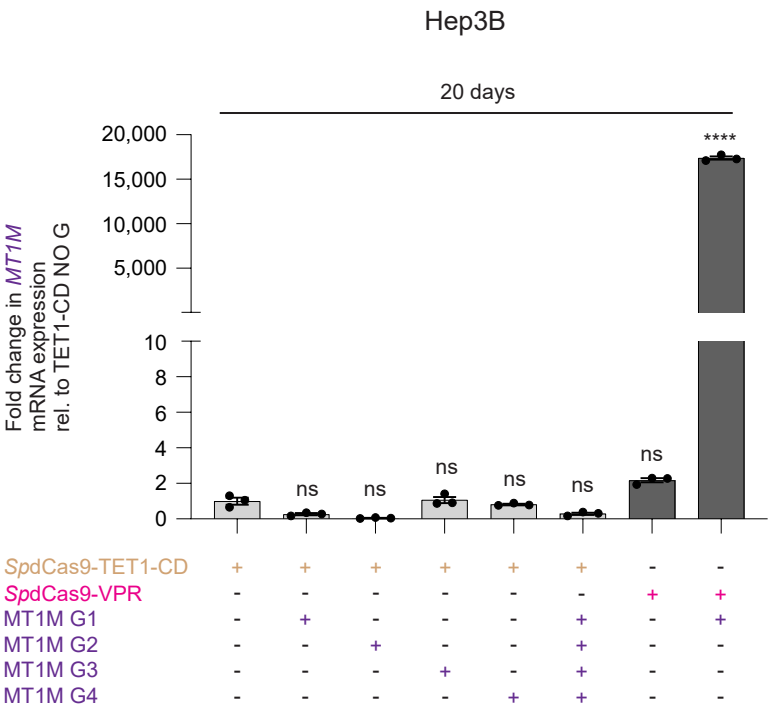

Supplement: Supplementary file 8 — Additional file 8: Figure S7. Related to Fig. 5. Lentiviral transduction of SpdCas9-TET1-CD or SpdCas9-VPR targeting HHIP or MT1M genes in Hep3B cells. A Schematic representation of the “all-in-one” lentiviral vectors, SpdCas9-TET1-CD and SpdCas9-VPR, for the constitutive co-expression of a gRNA and TET1-CD or VPR, C-terminally fused to SpdCas9. B Fold change in HHIP mRNA expression assessed by qRT-PCR at 18, 30, and 40 days in Hep3B cells constitutively co-expressing SpdCas9-TET1-CD and the combination of four gRNAs (MIX 4G) targeting HHIP promoter. C Fold change in MT1M mRNA expression assessed by qRT-PCR at 20 days in Hep3B cells lentivirally co-transduced with SpdCas9-TET1-CD and individual gRNAs (G1, G2, G3, and G4) or the MIX 4G; or with SpdCas9-VPR and the most potent gRNA (G1). Data presented as means ± SEM (n = 3), compared to SpdCas9-TET1-CD NO G, and P-values were determined by two-way ANOVA with Šídák's multiple comparisons test for panel (B), and by two-way ANOVA with Dunnett's multiple comparisons test for panel (C) (****P < 0.0001). SpdCas9 Streptococcus pyogenes deactivated Cas9 protein adopted for epigenome engineering, TET1-CD Ten-Eleven Translocation methylcytosine dioxygenase 1-catalytic domain, VPR VP64, p65, Rta, hU6 RNA polymerase III promoter for human U6 snRNA, hUbC human ubiquitin C promoter, 3xFLAG three tandem FLAG® epitope tags, followed by an enterokinase cleavage site, SV40NLS nuclear localization signal of SV40 (simian virus 40) large T antigen, T2A 2A peptide from Thosea asigna virus capsid protein, cleavable linker, PuroR puromycin N-acetyltransferase gene that confers resistance to puromycin, MIX 4G combination of four gRNAs, ns not significant. [file 13148_2023_1482_MOESM8_ESM.pdf]

Supplementary Figure S8

Hep3B

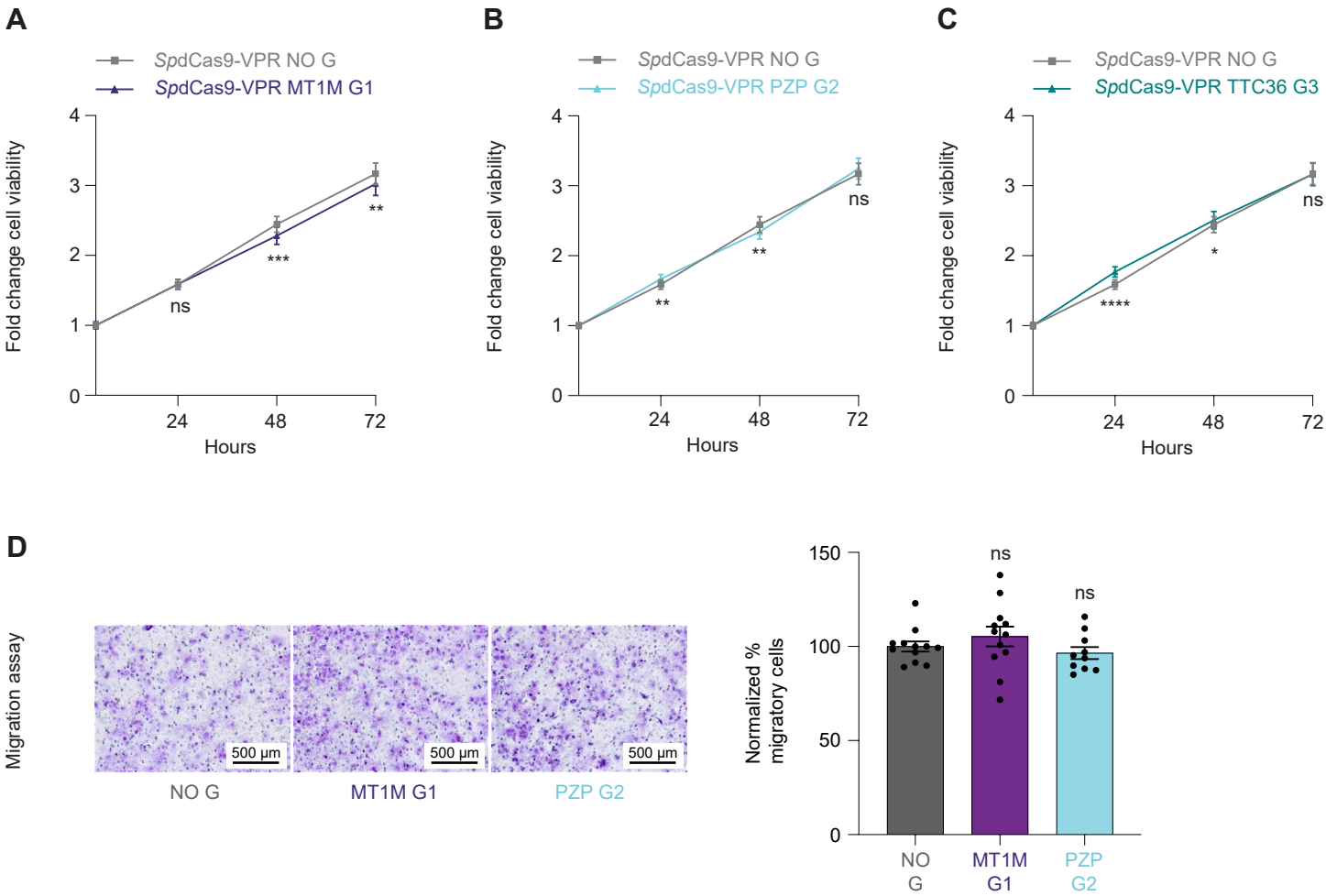

Supplement: Supplementary file 9 — Additional file 9: Figure S8. Related to Fig. 7. Phenotypic reprogramming in Hep3B HCC cells lentivirally transduced with SpdCas9-VPR targeting and upregulating MT1M with G1, PZP with G2, TTC36 with G3, or with NO G as control. A-C Cell viability determined using a luminescence assay (CellTiter-Glo®). Data shown as fold change compared to SpdCas9-VPR NO G at 24, 48, and 72 h, presented as means ± SEM (n = 3), and P-values were determined by unpaired t-test with Welch's correction (From left to right: (A), ***P = 0.0001, **P = 0.0056; (B), **P = 0.0024, **P = 0.0011; and (C), ****P < 0.0001, *P = 0.0310). D Cell migration assessed by the Boyden chamber assay. Data normalized to SpdCas9-VPR NO G, presented as means ± SEM (n = 3), and P-values were determined by unpaired t-test. ns not significant. [file 13148_2023_1482_MOESM9_ESM.pdf]
