# Supplementary material for: Epigenetic reactivation of tumor suppressor genes with CRISPRa technologies as precision therapy for hepatocellular carcinoma
Source: Clin Epigenetics. 2023 Apr 29;15:73. doi: 10.1186/s13148-023-01482-0 (PMC10149030; doi:10.1186/s13148-023-01482-0)
Supplement: Supplementary file 4 — Additional file 4: Figure S4 (part 1). Related to Figs. 3–7. A Off-target analysis of the ten TSGs reactivated in this study, using the most potent gRNAs. The potential off-target genes (MMP11, MOCOS, PIR, SEMA5A, TMEM14C, HRH1, NACC2, NONO, PPA1, RBM39, NKAIN3, and ABCC3) were found in proximity to genomic regulatory regions. [file 13148_2023_1482_MOESM4_ESM.pdf]

Supplementary Figure S4 (part 1)

A

Targeted gene	gRNA name	gRNA sequence	Mismatches	Potential off-target genes in regulatory regions
CDKN2A (-) Chr9	G1	CTTCCGGCTGGTGCCCCCGG	3	*MMP11*
CDKN2A (-) Chr9	G2	CCAACCTGGGGCGACTTCAG	3	*MOCOS*
CDKN2A (+) Chr9	G3	CGCCGTGAGCGAGTGCTCGG	3	*PIR*, *SEMA5A*, *TMEM14C*
CDKN2A (-) Chr9	G4	CCTTGCCTGGAAAGATACCG		none
CDKN2A (-) Chr9	G5	ATTTGAGGGACAGGGTCGGA		none
CPS1 (+) Chr2	G2	AAAGAGCAAACCTGGGTGTG	3	*HRH1*, *NACC2*, *NONO*
HHIP (+) Chr4	G4	AGGAACAGAAACGGCGACGG	3	*PPA1*, *RBM39*
miR-122-5p (+) Chr18	G2	CTGGATCCCATAAAGGGAGA		none
MT1E (+) Chr16	G1	CGAGCGAACGGGCTCCAAAG		none
MT1M (+) Chr16	G1	GCGGTGCGAACCCAGCCGGG	3	*NKAIN3*
PTGR1 (-) Chr9	G2	GGTGTTCAGCAGGCGGACTG	3	*ABCC3*
PZP (-) Chr12	G2	AAAAGGGTCATTACTCTCAG		none
TMEM106A (+) Chr17	G1	TCGGGTAACGTTTGAAGAGC		none
TTC36 (+) Chr11	G3	GGGAGACACTCCTCACGAAG		none
