# Supplementary material for: Epigenetic reactivation of tumor suppressor genes with CRISPRa technologies as precision therapy for hepatocellular carcinoma
Source: Clin Epigenetics. 2023 Apr 29;15:73. doi: 10.1186/s13148-023-01482-0 (PMC10149030; doi:10.1186/s13148-023-01482-0)
Supplement: Supplementary file 10 — Additional file 10: Table S1. Related to Figs. 3–7 and Figures S4-S8. gRNAs designed for gene reactivation by CRISPRa technology. Details include gRNA and PAM sequences, the DNA strand targeted, the distance from the TSS, and the genomic annotations. [file 13148_2023_1482_MOESM10_ESM.pdf]

Supplementary Table S1. gRNAs designed for gene reactivation by CRISPRa technology. The genomic annotations for each designed gRNA are from the Genome Reference Consortium Human Build 38 (GRCh38/hg38). The distance from the TSS is expressed in base pairs. PAM protospacer-adjacent motif, Chr chromosome, - negative or antisense DNA strand, + positive or sense DNA strand, TSS transcription start site.

Target gene	gRNA name	gRNA sequence	PAM sequence	DNA strand targeted	Distance from the TSS	Genomic annotation (start)	Genomic annotation (end)
CDKN2A	G1	CTTCCGGCTGGTGCCCCCGG	GGG	Chr9 -	+9	21975105	21975124
CDKN2A	G2	CCAACCTGGGGCGACTTCAG	GGG	Chr9 -	+36	21975078	21975097
CDKN2A	G3	CGCCGTGAGCGAGTGCTCGG	AGG	Chr9 +	+119	21975015	21975034
CDKN2A	G4	CCTTGCCTGGAAAGATACCG	CGG	Chr9 -	+122	21974992	21975011
CDKN2A	G5	ATTTGAGGGACAGGGTCGGA	GGG	Chr9 -	+157	21974957	21974976
CPS1	G1	GGCCTAAGTTTGTCCTTCTG	AGG	Chr2 +	-69	210477613	210477632
CPS1	G2	AAAGAGCAAACCTGGGTGTG	GGG	Chr2 -	+7	210477669	210477688
CPS1	G3	AATTCATGAAGATTTAGCCG	AGG	Chr2 +	+56	210477738	210477757
CPS1	G4	TCCAGCTTAAGACTCACCAT	GGG	Chr2 -	+102	210477764	210477783
HHIP	G1	GCGAGAAGCGGTGACGTCAA	GGG	Chr4 +	+74	144646070	144646089
HHIP	G2	TTTTTAACTAGCGCGCGGGG	AGG	Chr4 -	+136	144646112	144646131
HHIP	G3	GCTGAGGTCTCCTCTGCCGG	CGG	Chr4 -	+209	144646185	144646204
HHIP	G4	AGGAACAGAAACGGCGACGG	CGG	Chr4 -	+277	144646253	144646272
miR-122-5p	G1	TTTATCGATTCAGTGGGGGA	GGG	Chr18 +	+391	58446160	58446179
miR-122-5p	G2	CTGGATCCCATAAAGGGAGA	GGG	Chr18 +	+437	58446225	58446244
miR-122-5p	G3	GAGCTGACTGGGGGTAAGTG	CGG	Chr18 +	+537	58446325	58446344
miR-122-5p	G4	AAGAACGGCCTGATCACTCA	GGG	Chr18 -	+606	58446375	58446394
MT1E	G1	CGAGCGAACGGGCTCCAAAG	GGG	Chr16 +	-97	56625576	56625595
MT1E	G2	GCTGCTCTATTTATAGACGG	AGG	Chr16 -	+93	56625746	56625765
MT1E	G3	AGTGAGACGAACAAGCAGTC	AGG	Chr16 -	+148	56625801	56625820
MT1E	G4	CGCAGAGCTTCCCTTACCAG	TGG	Chr16 -	+224	56625877	56625896
MT1M	G1	GCGGTGCGAACCCAGCCGGG	CGG	Chr16 +	-62	56632560	56632579
MT1M	G2	GCGGCTGCTTTTCTACTCAG	GGG	Chr16 -	+22	56632624	56632643
MT1M	G3	GCTCCACCACGCCGTCCGGG	TGG	Chr16 +	+32	56632654	56632673
MT1M	G4	AATGGGATTCCTAGGGCGCA	GGG	Chr16 -	+175	56632777	56632796
PTGR1	G1	TGAACGTGAAGCTAAGTCGT	TGG	Chr9 +	-529	111600385	111600404
PTGR1	G2	GGTGTTCAGCAGGCGGACTG	GGG	Chr9 -	-363	111600199	111600218
PTGR1	G3	CGGTGCTGGCTCCGATCCCT	GGG	Chr9 +	-240	111600096	111600115
PTGR1	G4	CGGCGGAAATATGCCCCGTG	GGG	Chr9 +	-137	111599993	111600012
PZP	G1	GTTGAAGGTTCCTTATCAGG	AGG	Chr12 -	-268	9208619	9208638
PZP	G2	AAAAGGGTCATTACTCTCAG	TGG	Chr12 +	-126	9208497	9208516
PZP	G3	TGGGGCTTGTTTGTACATAG	TGG	Chr12 -	-81	9208432	9208451
PZP	G4	GAGATTTATCCCTCACAATG	CGG	Chr12 -	+12	9208339	9208358
TMEM106A	G1	TCGGGTAACGTTTGAAGAGC	TGG	Chr17 +	-98	43211729	43211748
TMEM106A	G2	GCACTGAGTCAAAGCTTGAG	GGG	Chr17 +	+10	43211837	43211856
TMEM106A	G3	GAGAGTGGAATCGAAAATGC	GGG	Chr17 -	+66	43211873	43211892
TMEM106A	G4	TAGGTATAAAGGGTCTAGGG	AGG	Chr17 -	+126	43211933	43211952
TTC36	G1	ATCCAGCTTCAGCCTTAAGG	TGG	Chr11 -	-243	118527232	118527251
TTC36	G2	CCAAGTAGTCTGCCAAGCCA	GGG	Chr11 +	-200	118527295	118527314
TTC36	G3	GGGAGACACTCCTCACGAAG	GGG	Chr11 +	-131	118527364	118527383
TTC36	G4	TTTTGGAGTTGAGCACCATG	GGG	Chr11 +	-17	118527478	118527497
