# Supplementary material for: Epigenetic reactivation of tumor suppressor genes with CRISPRa technologies as precision therapy for hepatocellular carcinoma
Source: Clin Epigenetics. 2023 Apr 29;15:73. doi: 10.1186/s13148-023-01482-0 (PMC10149030; doi:10.1186/s13148-023-01482-0)
Supplement: Supplementary file 11 — Additional file 11: Table S2. Related to Figs. 2–7 and Figures S4-S7. TaqMan gene expression assays utilized for qRT-PCR experiments. [file 13148_2023_1482_MOESM11_ESM.pdf]

Supplementary Table S2. TaqMan gene expression assays utilized for qRT-PCR in this study. For each gene, the TaqMan Assay ID and reporter dye are listed. FAM 6-Carboxyfluorescein (6-FAM), MGB minor groove binder.

Gene	TaqMan Assay ID	Dye
BCO2	Hs00230564_m1	FAM-MGB
CDKN2A	Hs00923894_m1	FAM-MGB
CPS1	Hs00157048_m1	FAM-MGB
HHIP	Hs01011015_m1	FAM-MGB
hsa-miR-122-5p	002245	FAM-MGB
MT1E	Hs01938284_g1	FAM-MGB
MT1M	Hs00828387_g1	FAM-MGB
PSAT1	Hs00795278_mH	FAM-MGB
PTGR1	Hs00400932_m1	FAM-MGB
PZP	Hs00161140_m1	FAM-MGB
TMEM106A	Hs00376739_m1	FAM-MGB
TTC36	Hs01377099_m1	FAM-MGB
GAPDH	Hs99999905_m1	FAM-MGB
GUSB	Hs00939627_m1	FAM-MGB
PPIA	Hs04194521_s1	FAM-MGB
RNU6B	001093	FAM-MGB
ABCC3	Hs00978452_m1	FAM-MGB
HRH1	Hs00911670_s1	FAM-MGB
MMP11	Hs00968295_m1	FAM-MGB
MOCOS	Hs00215742_m1	FAM-MGB
NACC2	Hs00299236_m1	FAM-MGB
NKAIN3	Hs00543621_m1	FAM-MGB
NONO	Hs00939763_g1	FAM-MGB
PIR	Hs01125822_m1	FAM-MGB
PPA1	Hs00535680_g1	FAM-MGB
RBM39	Hs00863502_g1	FAM-MGB
SEMA5A	Hs01549381_m1	FAM-MGB
TMEM14C	Hs00977207_g1	FAM-MGB
